# Supplementary material for: CDK12 controls G1/S progression by regulating RNAPII processivity at core DNA replication genes
Source: EMBO Rep. 2019 Jul 25;20(9):e47592. doi: 10.15252/embr.201847592 (PMC6727028; doi:10.15252/embr.201847592)

CDK12 controls G1/S progression by regulating RNAPII processivity at core DNA replication genes

Anil Paul Chirackal Manavalan[1], Kveta Pilarova[1], Michael Kluge[2], Koen Bartholomeeusen[1,6], Michal Rajecky[1], Jan Oppelt[1], Prashant Khirsariya[4,5], Kamil Paruch[4,5], Lumir Krejci[3,5,7], Caroline C. Friedel[2], Dalibor Blazek[1,8]

TABLE OF CONTENTS:

1. Appendix Figure Legends –Page 2-5
2. Appendix Figure S1 – Page 6
3. Appendix Figure S2 – Page 7
4. Appendix Figure S3 – Page 8
5. Appendix Figure S4 – Page 9
6. Appendix Figure S5 – Page 10
7. Appendix Figure S6 – Page 11
8. Appendix Figure S7 – Page 12
9. Appendix Figure S8 – Page 13-14
10. Appendix Figure S9 – Page 15
11. Appendix Figure S10 – Page 16
12. Appendix Figure S11 – Page 17
13. Appendix Figure S12 – Page 18
14. Appendix Figure S13 – Page 19
15. Appendix Figure S14 – Page 20
16. Appendix Figure S15 – Page 21

Appendix Figure Legends

Appendix Figure S1. Preparation and characterization of AS CDK12 HCT116 cell line.

a, Sanger sequencing of WT CDK12 HCT116 clones.

b, Sanger sequencing of AS CDK12 HCT116 clones.

Appendix Figure S2. Genes that are more strongly down-regulated have a tendency towards more reduced occupancy of RNAPII at their 3'ends.

Metagene analysis of ChIP-seq occupancies of RNAPII as described in Fig. 5b, c on groups of genes with the indicated log2 fold-change of expression in nuclear RNA-seq.

Appendix Figure S3. P-Ser5 occupancy normalized to RNAPII shows no or very little changes across genes after CDK12 inhibition.

Metagene analysis as described in Fig. 5b, c of ChIP-seq P-Ser5 occupancies normalized to RNAPII.

Appendix Figures S4. The shift of P-Ser2 into the gene body is most pronounced in strongly down-regulated genes.

Metagene analysis as described in Fig. 5b, c of ChIP-seq P-Ser2 on groups of genes with the indicated log2 fold-change of expression in nuclear RNA-seq.

Appendix Figures S5. P-Ser2 occupancy normalized to RNAPII shows small but highly significant changes after CDK12 inhibition.

Metagene analysis as described in Fig. 5b, c of ChIP-seq P-Ser2 occupancies normalized to RNAPII.

Appendix Figure S6. SPT6 travels together with RNAPII on genes independently of CDK12 kinase activity.

Metagene analysis as described in Fig. 5b, c of ChIP-seq SPT6 profiles normalized to RNAPII.

Appendix Figure S7. Examples of genes whose transcription processivity and expression is dependent on the CDK12 kinase activity.

a, b, c, Examples of genes whose transcription processivity and expression is dependent on the CDK12 kinase activity. Nuclear RNA-seq data and RNAPII, P-Ser2, P-Ser5 and SPT6 ChIP-seq data for *SWSWAP* (**a**), *TOPBP1* (**b**) and *MCM10* (**c**) as described in Figs. 5d, e.

Appendix Figure S8. CDK12 inhibition results in transcript shortening of a subset of genes.

a, Differentially used down-regulated exons are predominantly present at gene 3´ends. Graph shows the distribution of relative exon positions as described in Fig. 6b of differentially used down-regulated exons (according to DEXSeq, log2 fold-change ≤-1, p≤0.01, n=3473 genes).

b, Differentially used up-regulated exons are predominantly present at gene 5´ends. Graph shows the distribution of relative exon positions as described in Fig. 6b of differentially used up-regulated exons (according to DEXSeq, log2 fold-change≥1, p≤0.01, n=2017 genes).

c, Inhibition of CDK12 kinase activity results in shorting of a subset of transcripts. Box plot shows the relative position of all exons (n=282614 exons) in comparison to exons identified as either up-regulated (log2 fold-change≥1, p≤0.01, n=2017 genes) or down-regulated (log2 fold-change≤-1, p≤0.01, n=3473 genes) by DEXSeq. n=3 replicates.

d, In down-regulated genes without a significantly differentially used exons, the exons close to 5´ and 3´ends also tend to be weakly up- and down-regulated, respectively. Box plots show the log2 fold-change in exon usage after CDK12 inhibition determined by DEXSeq for exons in genes without differentially used exons (p≥0.01 for all exons). Exons were grouped into deciles according to their relative exon position. n=3 replicates.

e, f, Genes with up- or down-regulated exons (at least one exon with log2 fold-change ≥1 or ≤-1, respectively, p≤0.01) show similar shifts of RNAPII and P-Ser2 occupancy in comparison to genes without differentially used exons (p≥0.01 for all exons). Metagene analyses of RNAPII (**e**) and P-Ser2 (**f**) ChIP-seq data as described in Fig. 5b, c. P-Ser2 occupancy is normalized to RNAPII occupancy.

g, Distribution of exon usage changes in genes not down-regulated but with significantly regulated exons shows a similar trend as for the down-regulated genes. Box plot shows log2 fold-changes in exon usage after CDK12 inhibition determined by DEXSeq for genes not down-regulated. Exons were grouped into deciles according to their relative exon position. n=3 replicates.

Appendix Figure S9. CDK12 kinase activity is required for optimal transcription of long genes.

a, b, Longer genes tend to have a larger fraction of differentially used exons. The same analysis as in the Fig. 7a using only down-regulated (**a**) or upregulated (**b**) exons, respectively. n=3 replicates.

Appendix Figures S10-S12. Longer genes show stronger changes in RNAPII, P-Ser2 and P-Ser5 ChIP-seq occupancy after CDK12 inhibition. Metagene analysis as described in Fig. 5b, c of RNAPII (S10), P-Ser2 (S11) and P-Ser5 (S12) ChIP-seq on groups of genes with the indicated length.

Appendix Figure S13 and S14. Genes with shortened transcripts have reduced RNAPII occupancy at 3′ ends and show a shift of the P-Ser2 signal towards gene bodies. Metagene analysis as described in Fig. 5b, c of RNAPII (S13) and P-Ser2 (S14) ChIP-seq occupancies on groups of genes with the indicated changes in their transcript length. Absolute Δ90% distance = 90% distance in control - 90% distance in CDK12-inhibited cells (positive values indicate shortening of transcripts in CDK12-inhibited cells). Relative Δ90% = absolute Δ90% divided by gene length.

Appendix Figure S15. CDK12 kinase activity is required for optimal transcription of long, poly(A)-signal-rich genes.

a, Gene length and abundance of canonical poly(A) signals are correlated. Length and number of canonical poly(A) signal sequences (AATAAA, ATTAAA) in protein coding genes is plotted against each other. rho=Spearman rank correlation coefficient.

b, Presence of poly(A) signals contributes to the shortening of transcripts. Box plots show the difference in the 10, 50 and 90% distance divided by gene length between control and CDK12 inhibited cells. Genes were grouped into quantiles according to the number of poly(A) signals (AATAAA, ATTAAA) per kilobase (kb) of gene length. n=3 replicates.

A

WT CDK12 HCT116 cells

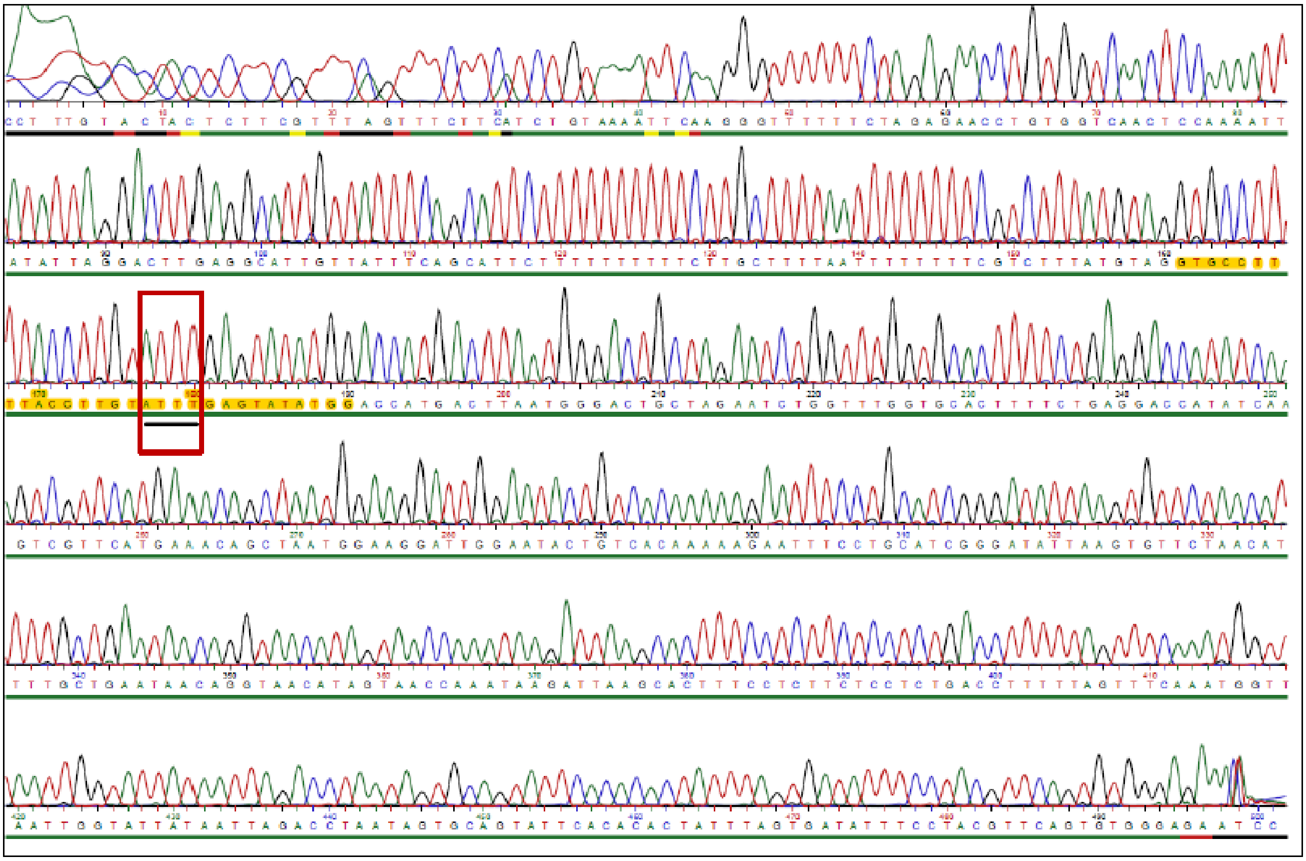

B

AS CDK12 HCT116 cells

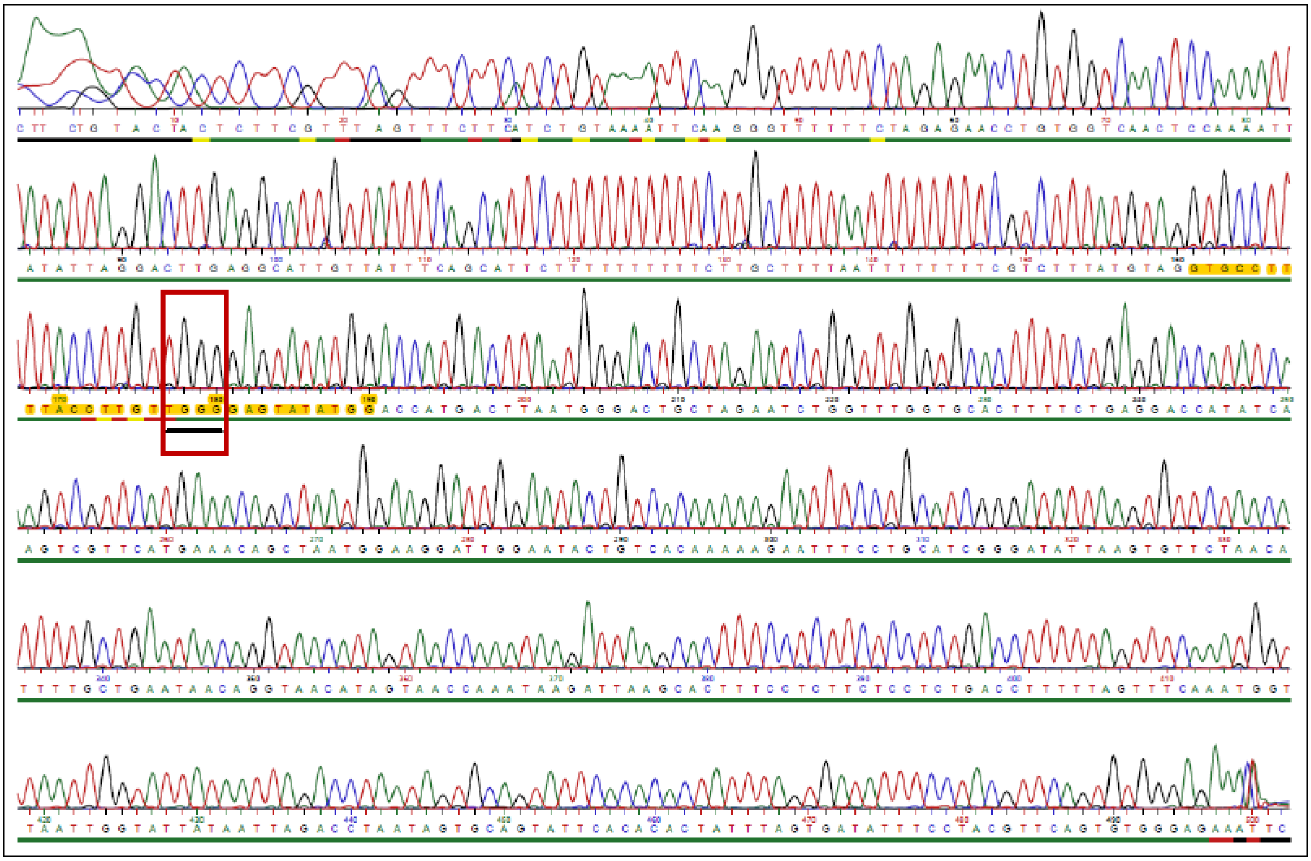

Appendix Fig. S2

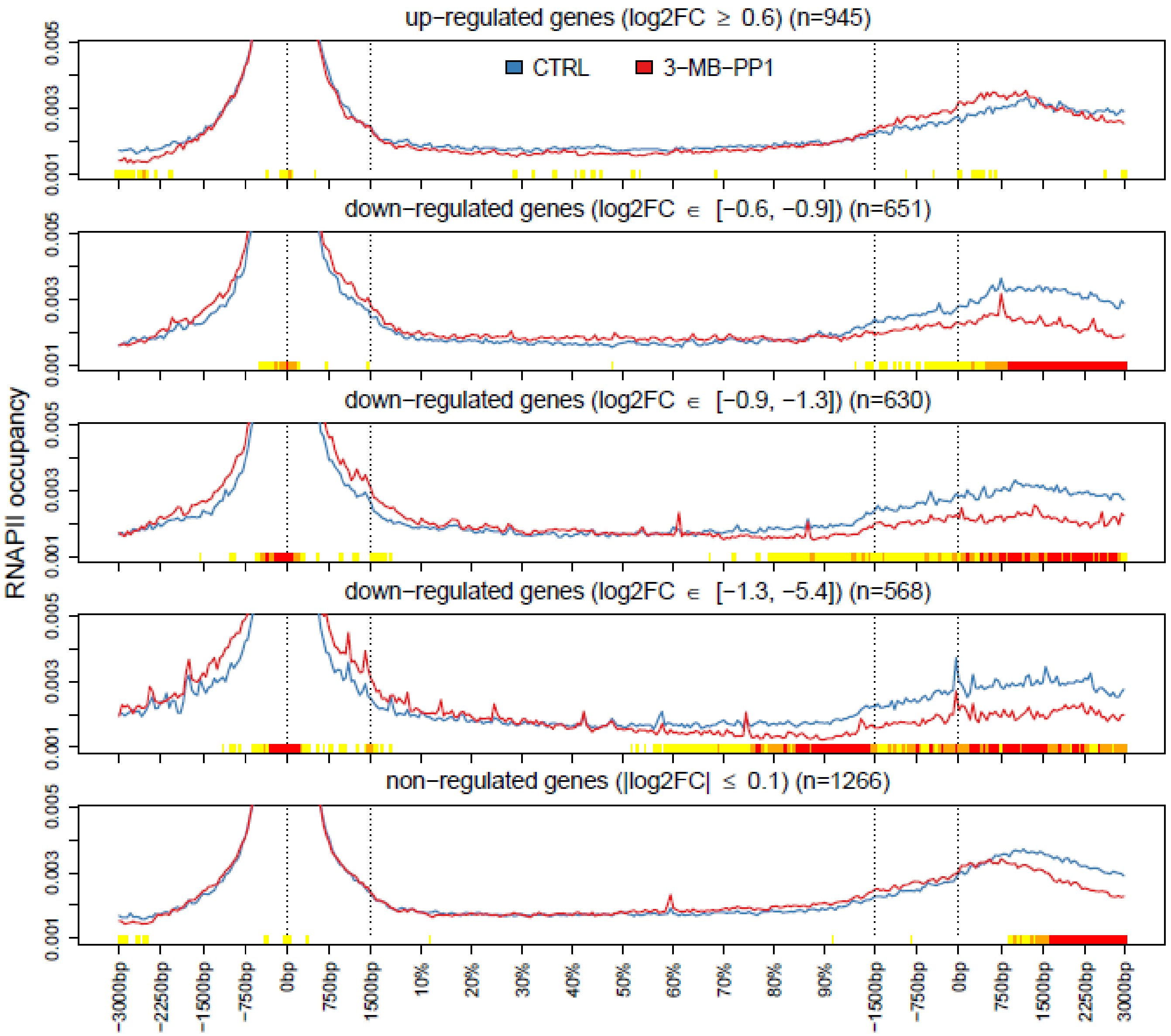

Appendix Fig. S3

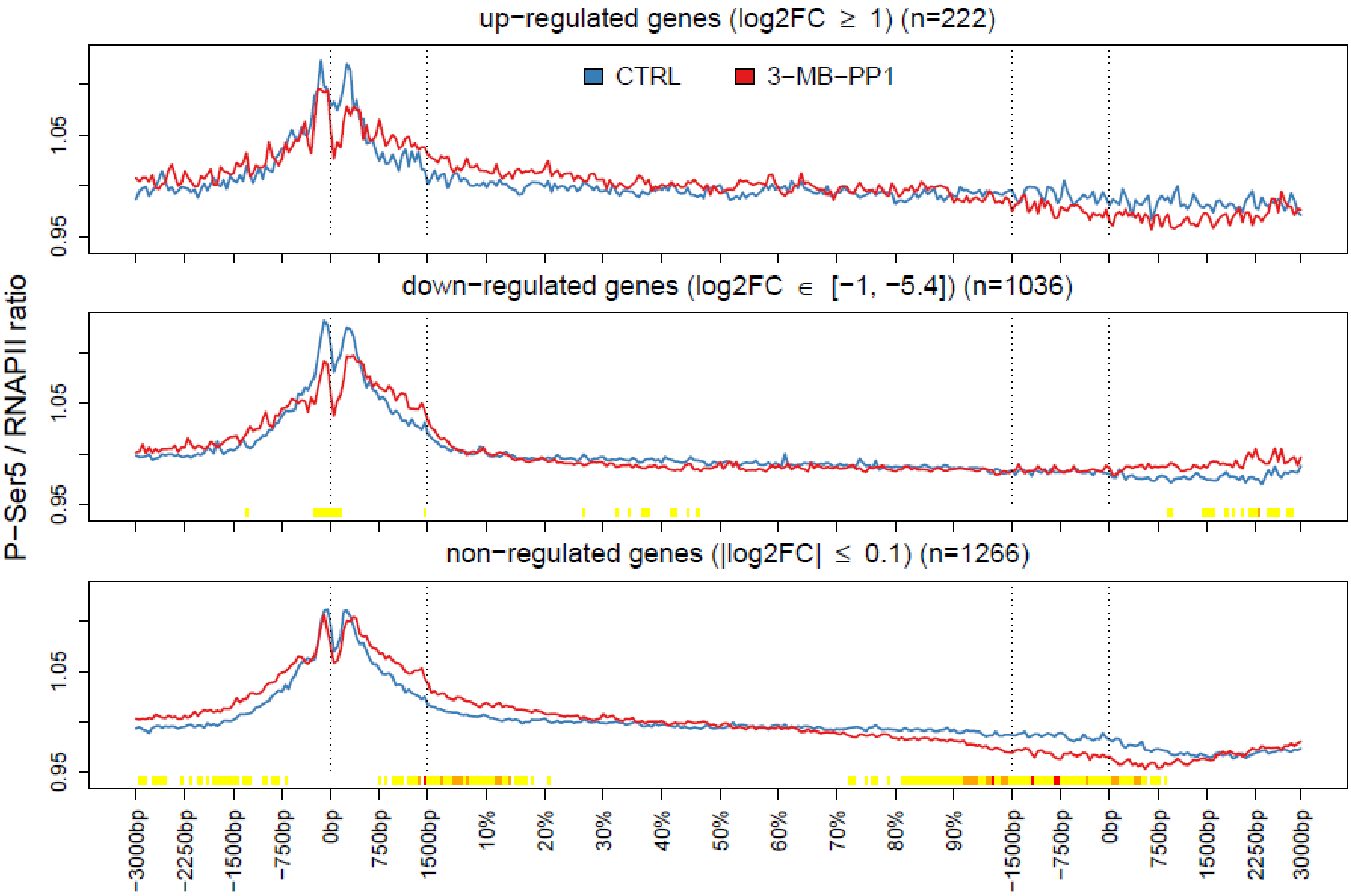

Appendix Fig. S4

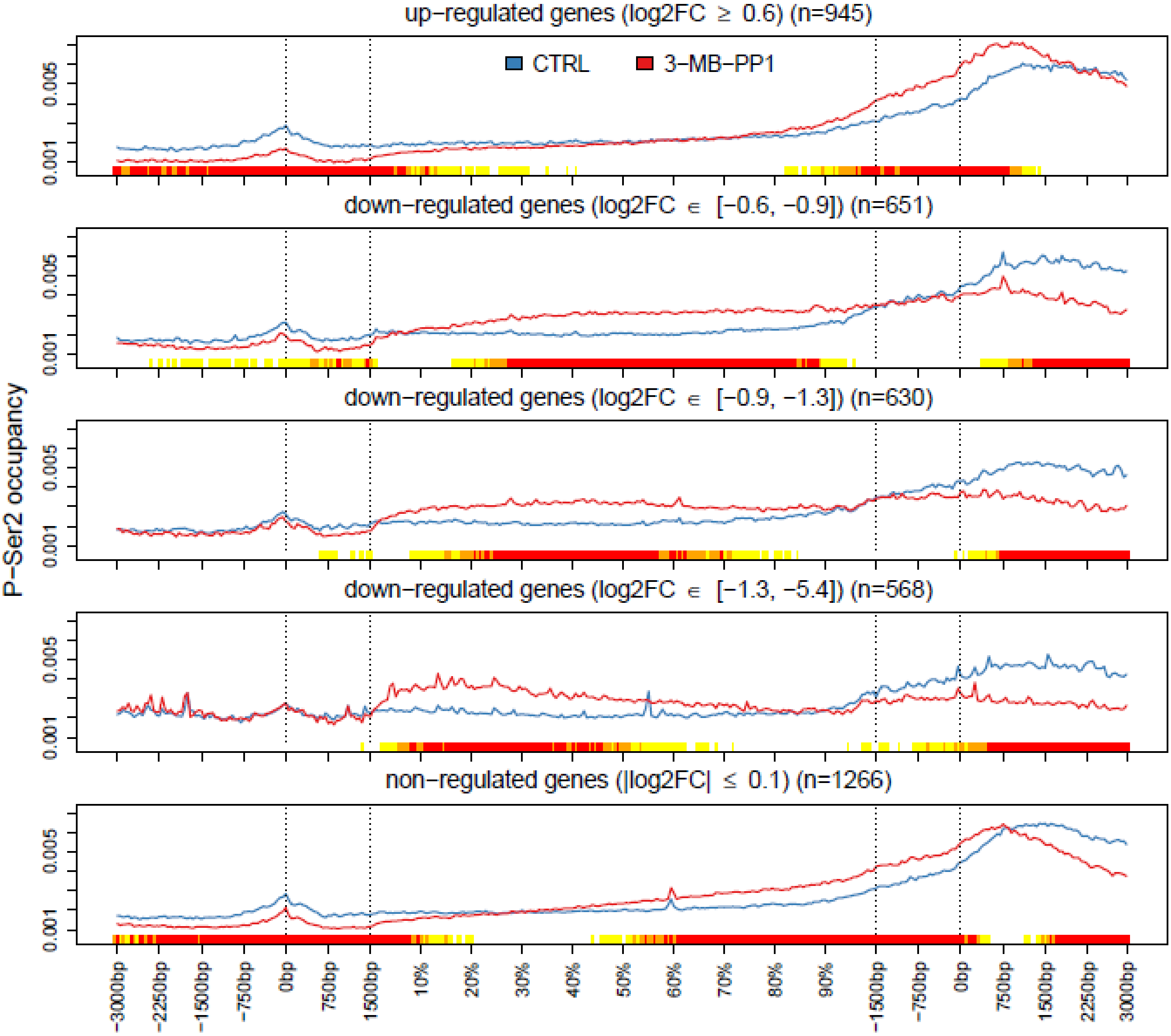

Appendix Fig. S5

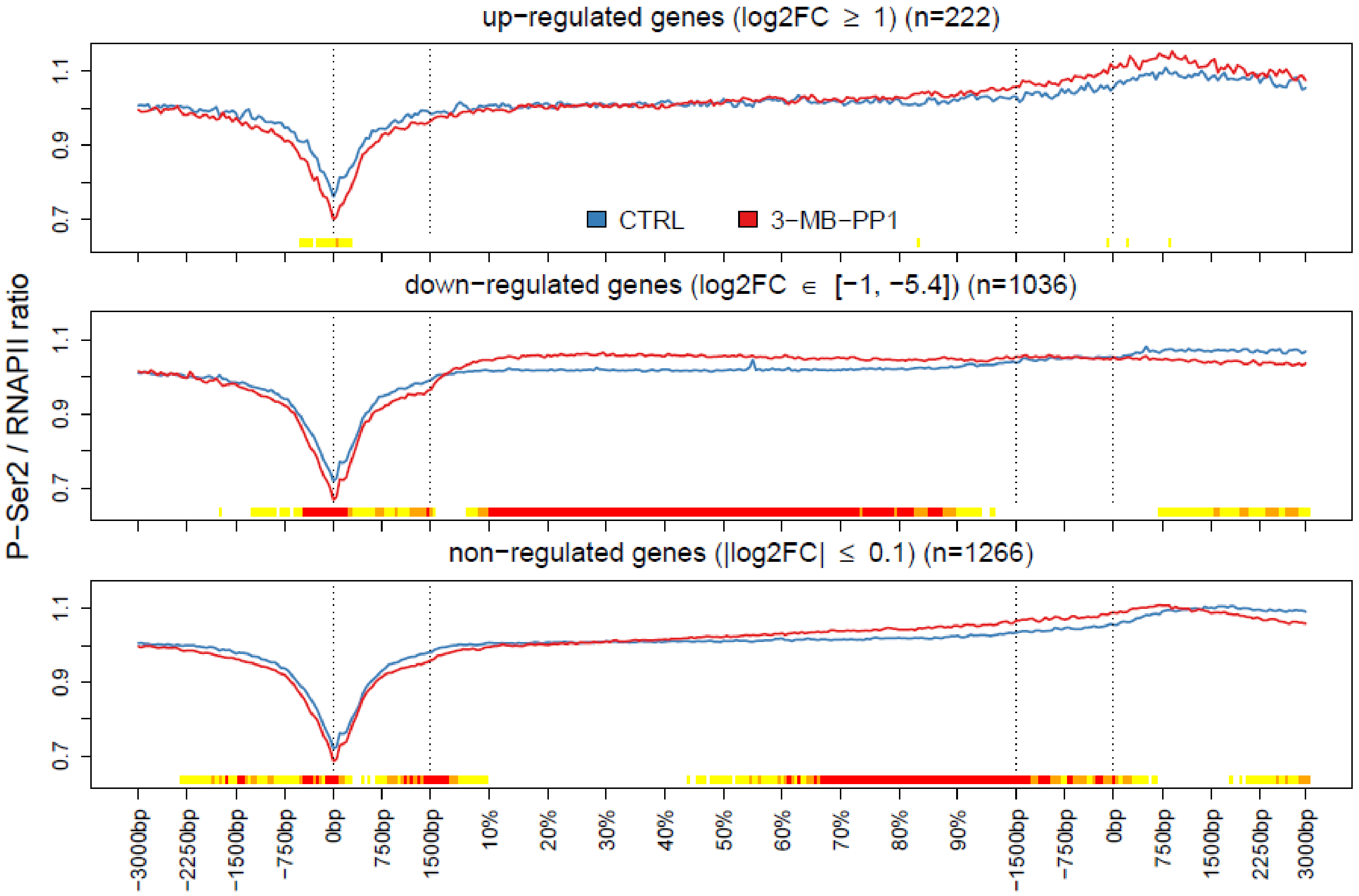

Appendix Fig. S6

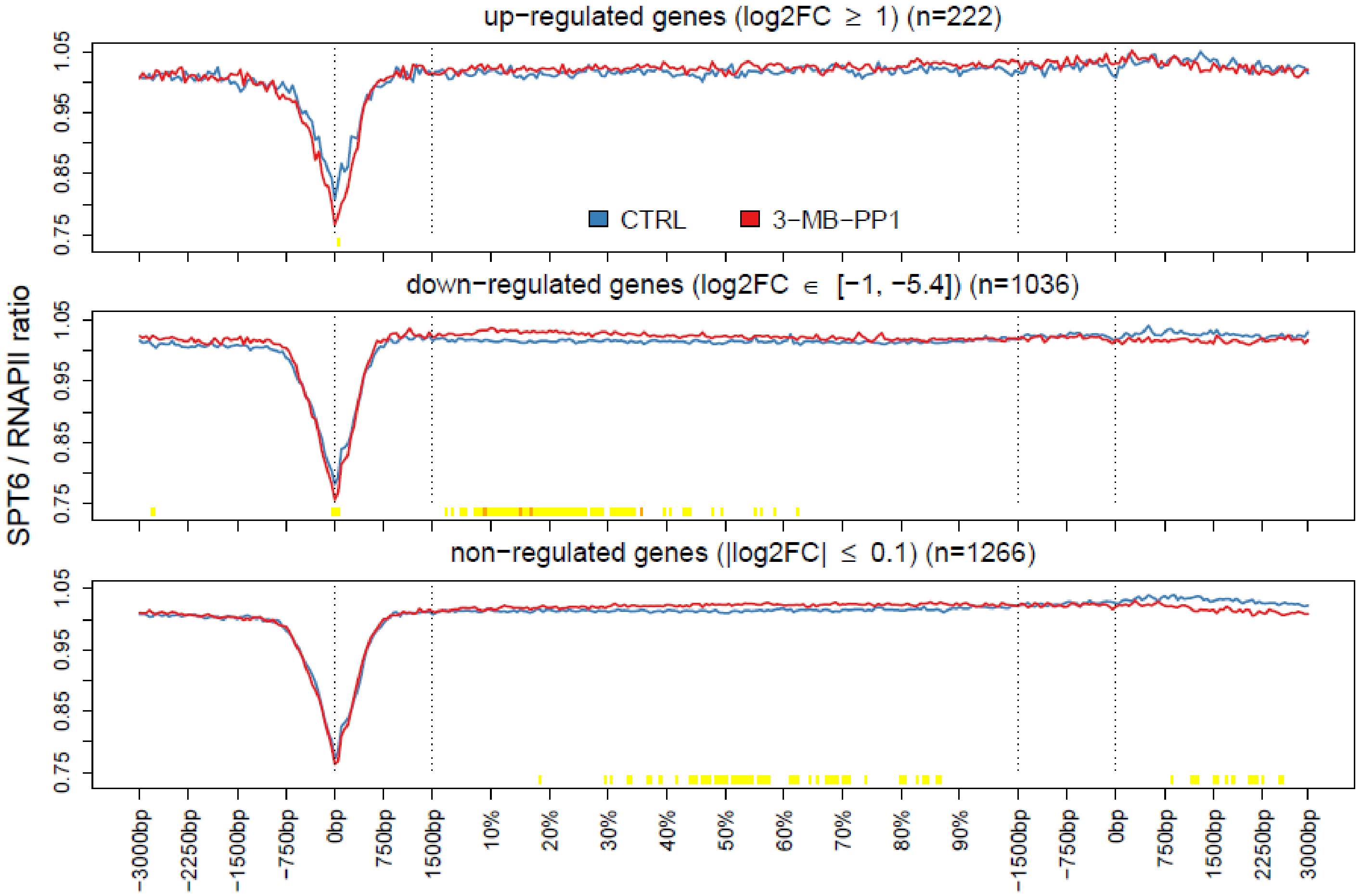

Appendix Fig. S7

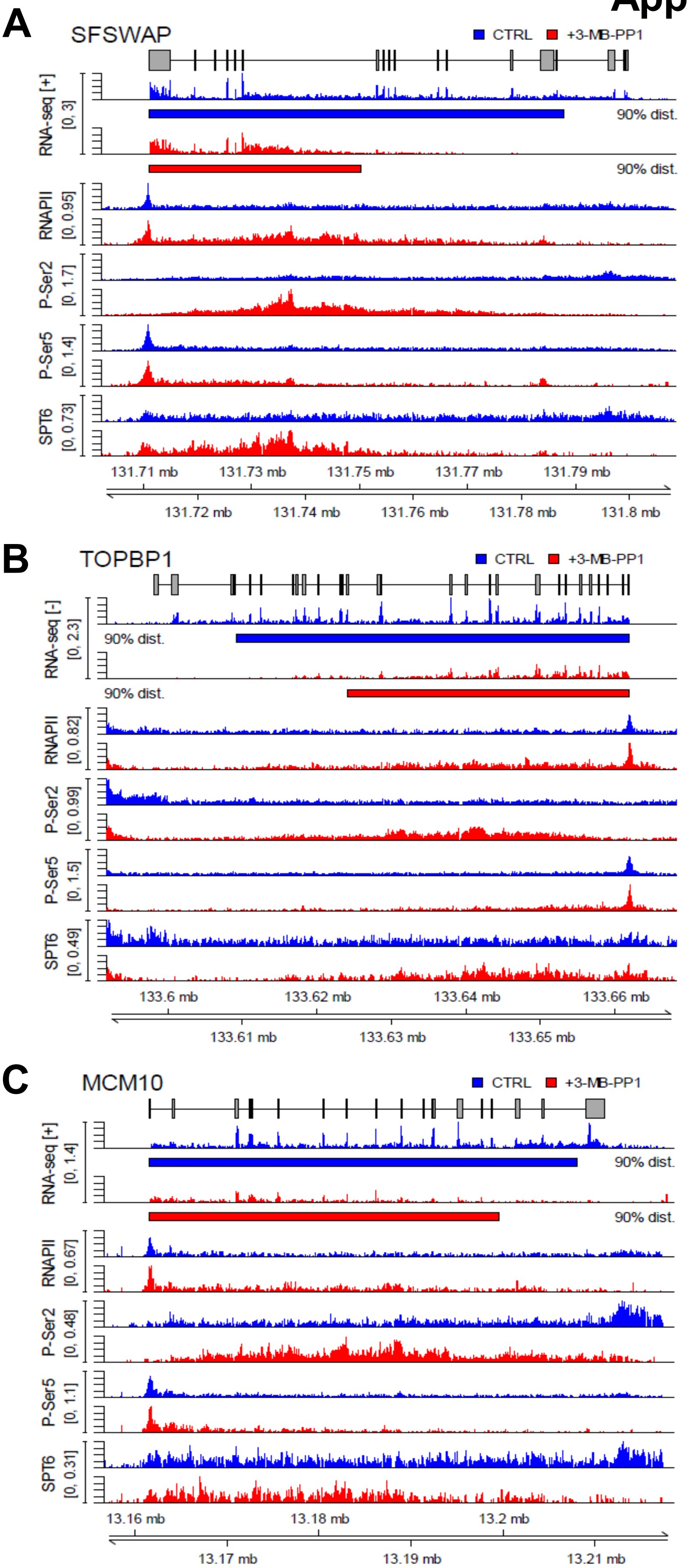

Appendix Fig. S8

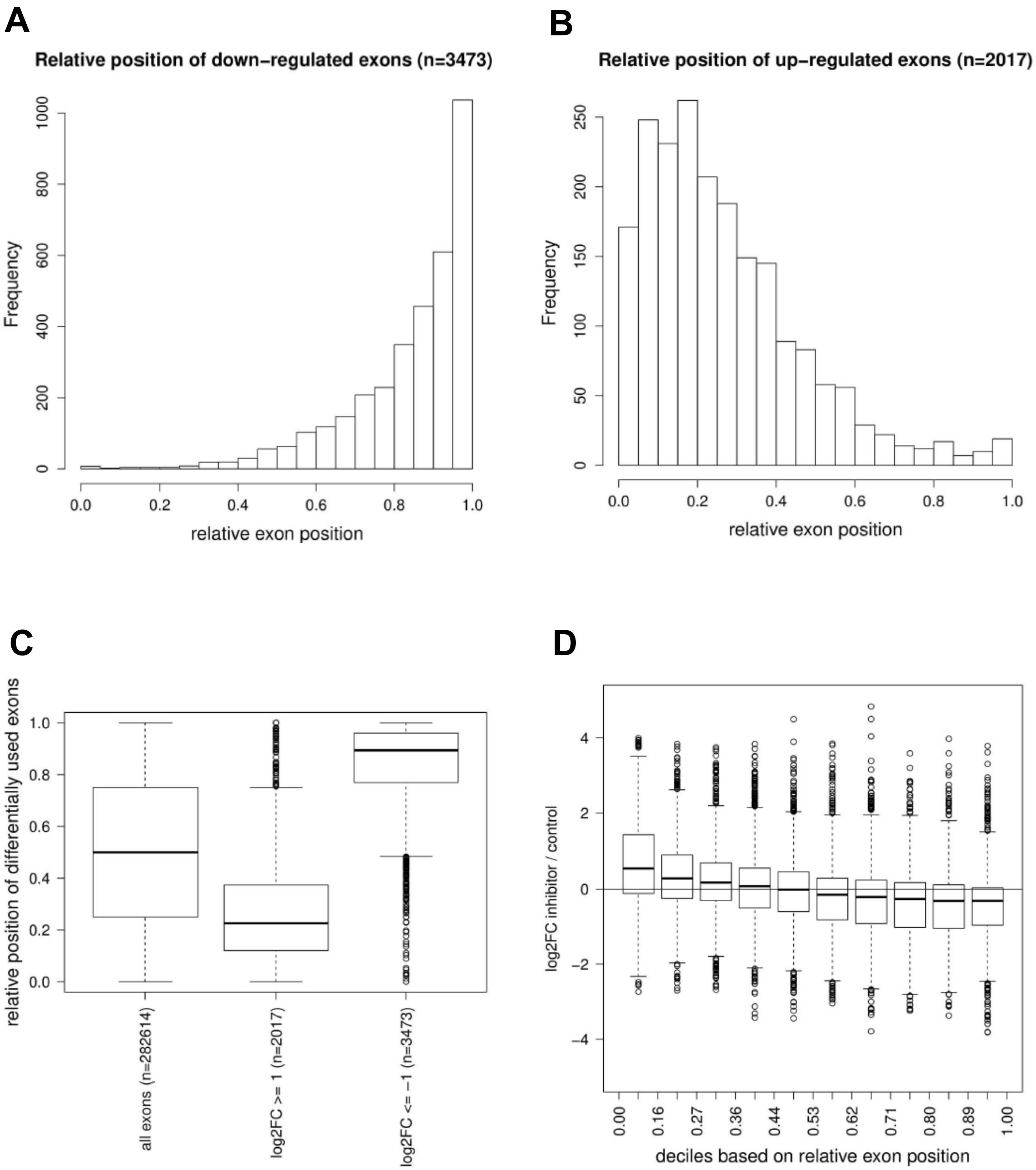

E

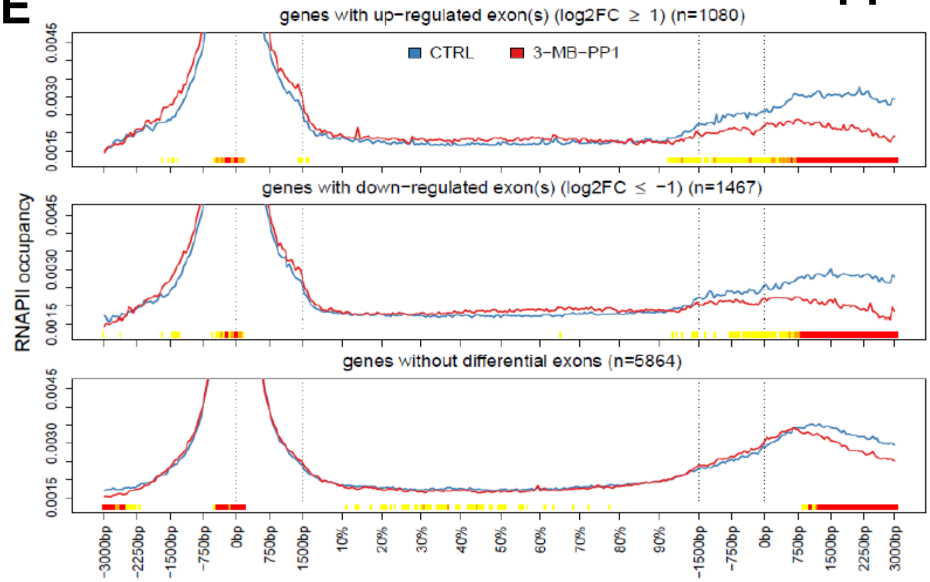
genes with up−regulated exon(s) (log2FC ≥ 1) (n=1080)
CTRL
3−MB−PP1
genes with down−regulated exon(s) (log2FC ≤ −1) (n=1467)
genes without differential exons (n=5864)
RNAPII occupancy
0.0015
0.0030
0.0045
−3000bp
−2250bp
−1500bp
−750bp
0bp
750bp
1500bp
10%
20%
30%
40%
50%
60%
70%
80%
90%
−1500bp
−750bp
0bp
750bp
1500bp
2250bp
3000bp

F

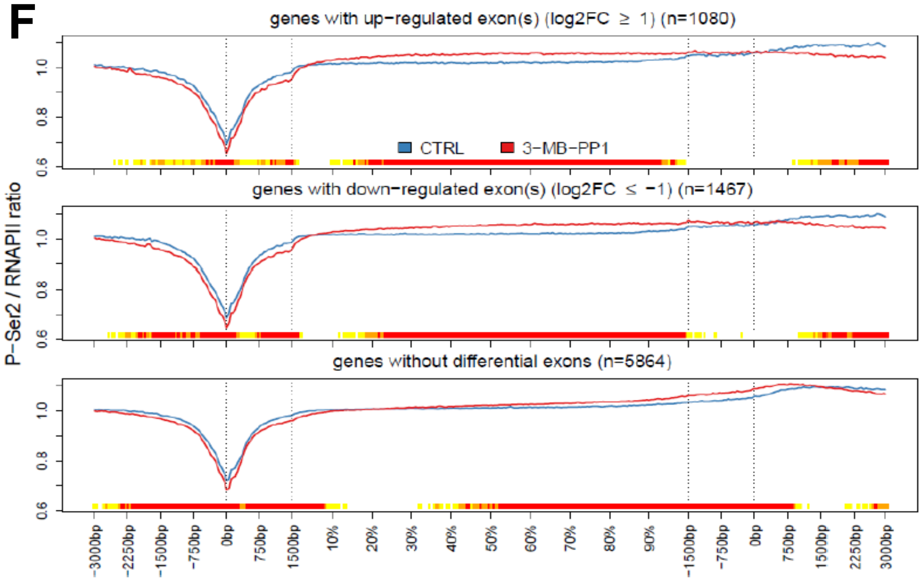
genes with up−regulated exon(s) (log2FC ≥ 1) (n=1080)
CTRL
3−MB−PP1
genes with down−regulated exon(s) (log2FC ≤ −1) (n=1467)
genes without differential exons (n=5864)
P−Ser2 / RNAPII ratio
0.6
0.8
1.0
−3000bp
−2250bp
−1500bp
−750bp
0bp
750bp
1500bp
10%
20%
30%
40%
50%
60%
70%
80%
90%
−1500bp
−750bp
0bp
750bp
1500bp
2250bp
3000bp

G

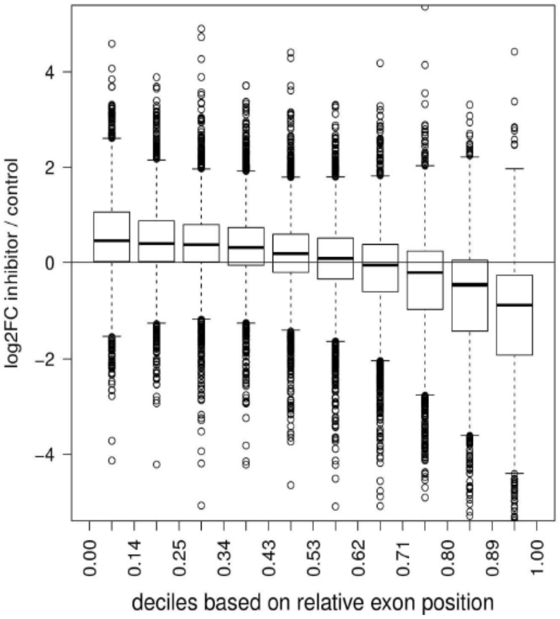
log2FC inhibitor / control
4
2
0
−2
−4
0.00
0.14
0.25
0.34
0.43
0.53
0.62
0.71
0.80
0.89
1.00
deciles based on relative exon position

Appendix Fig. S9

A

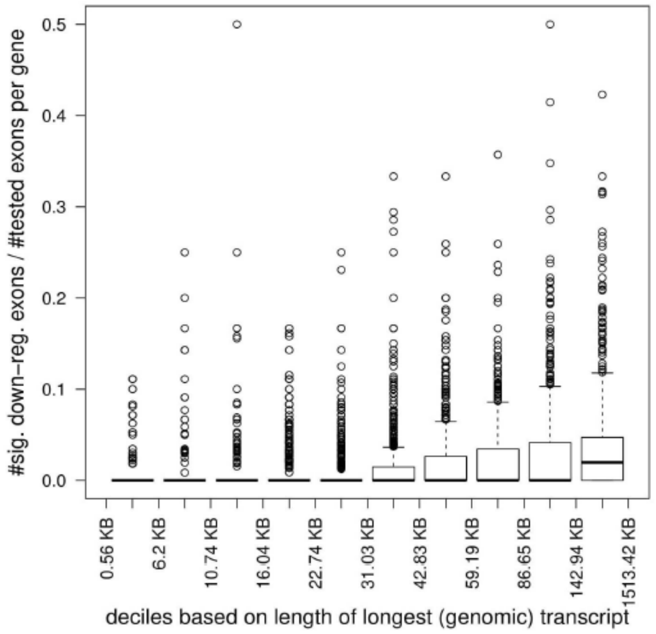

B

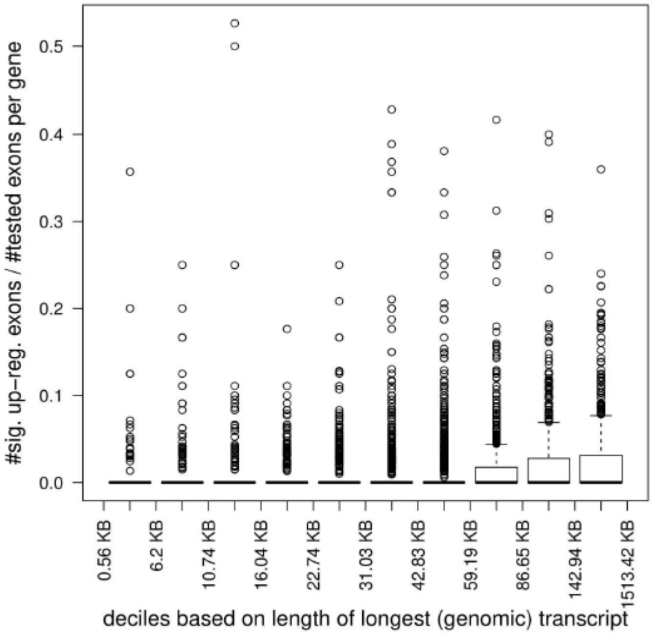

Appendix Fig. S10

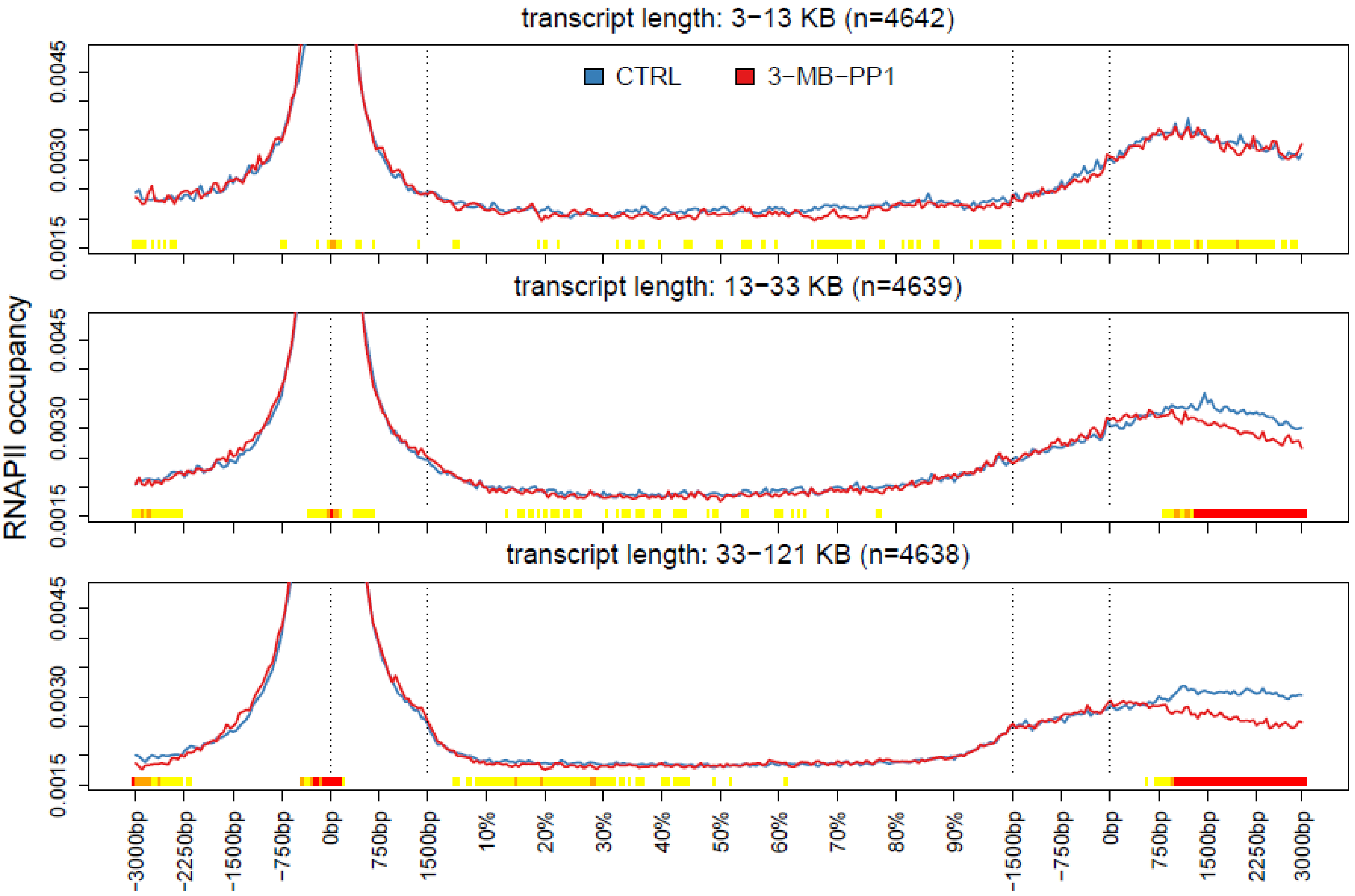

Appendix Fig. S11

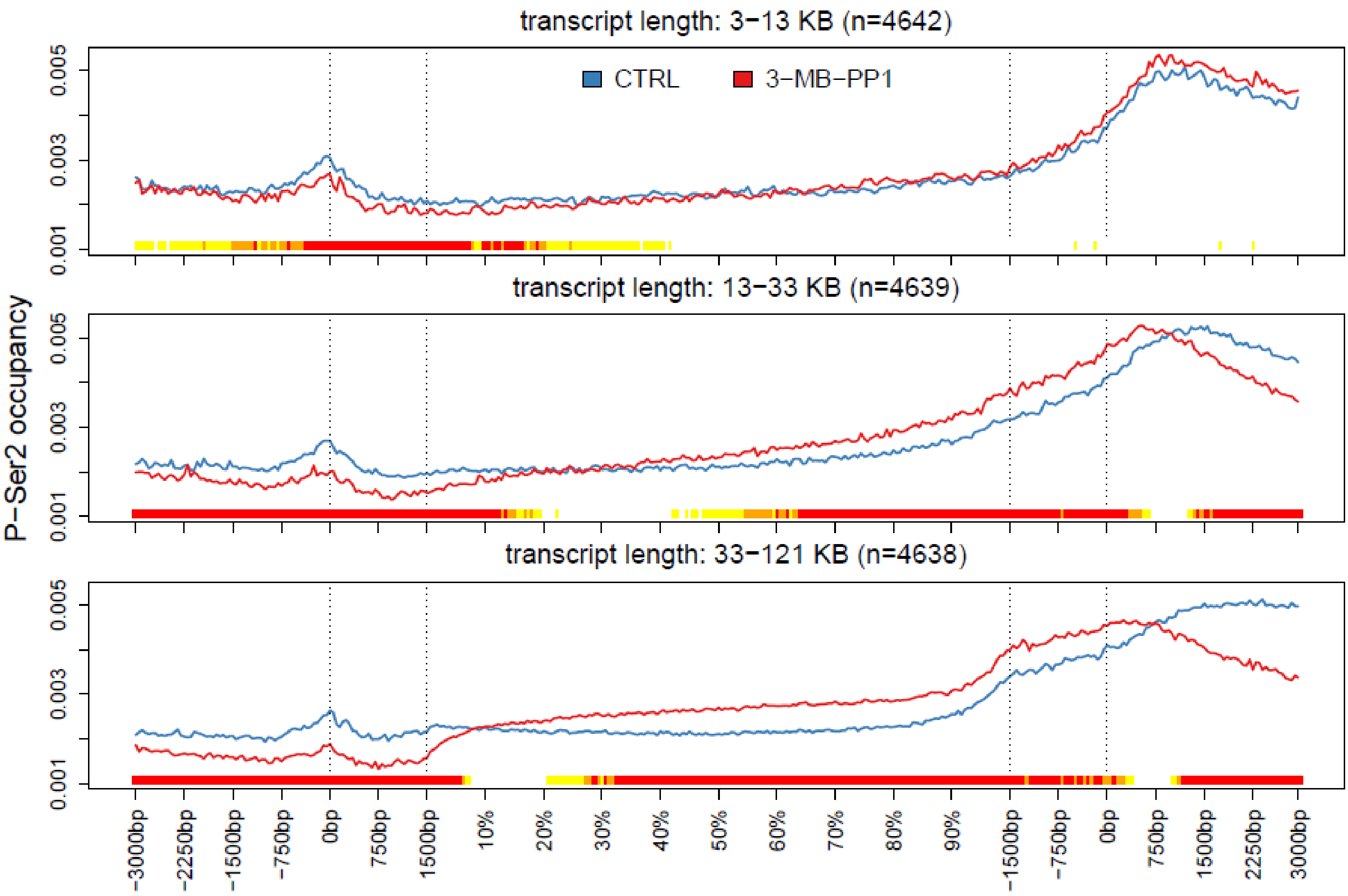

Appendix Fig. S12

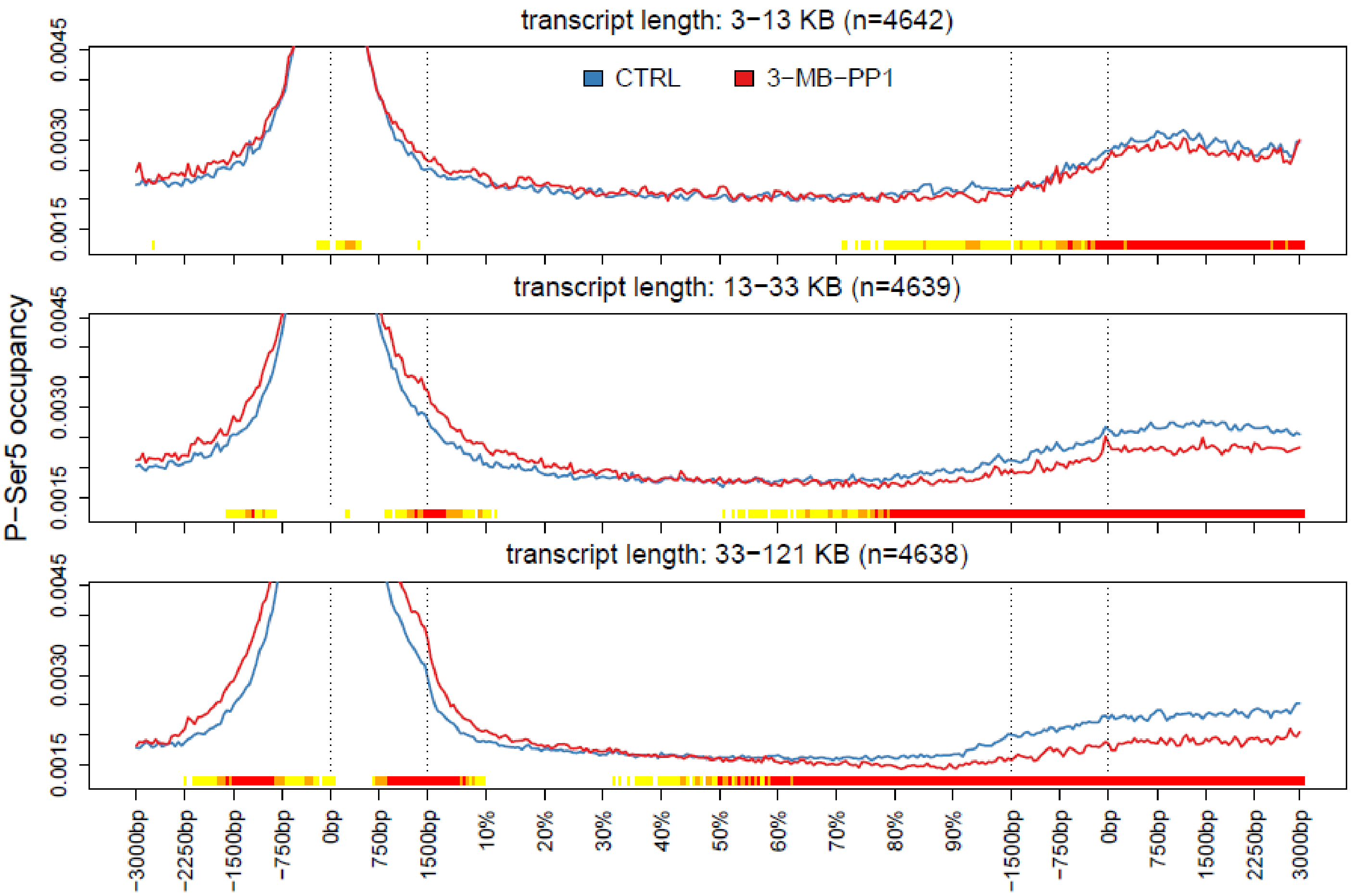

Appendix Fig. S13

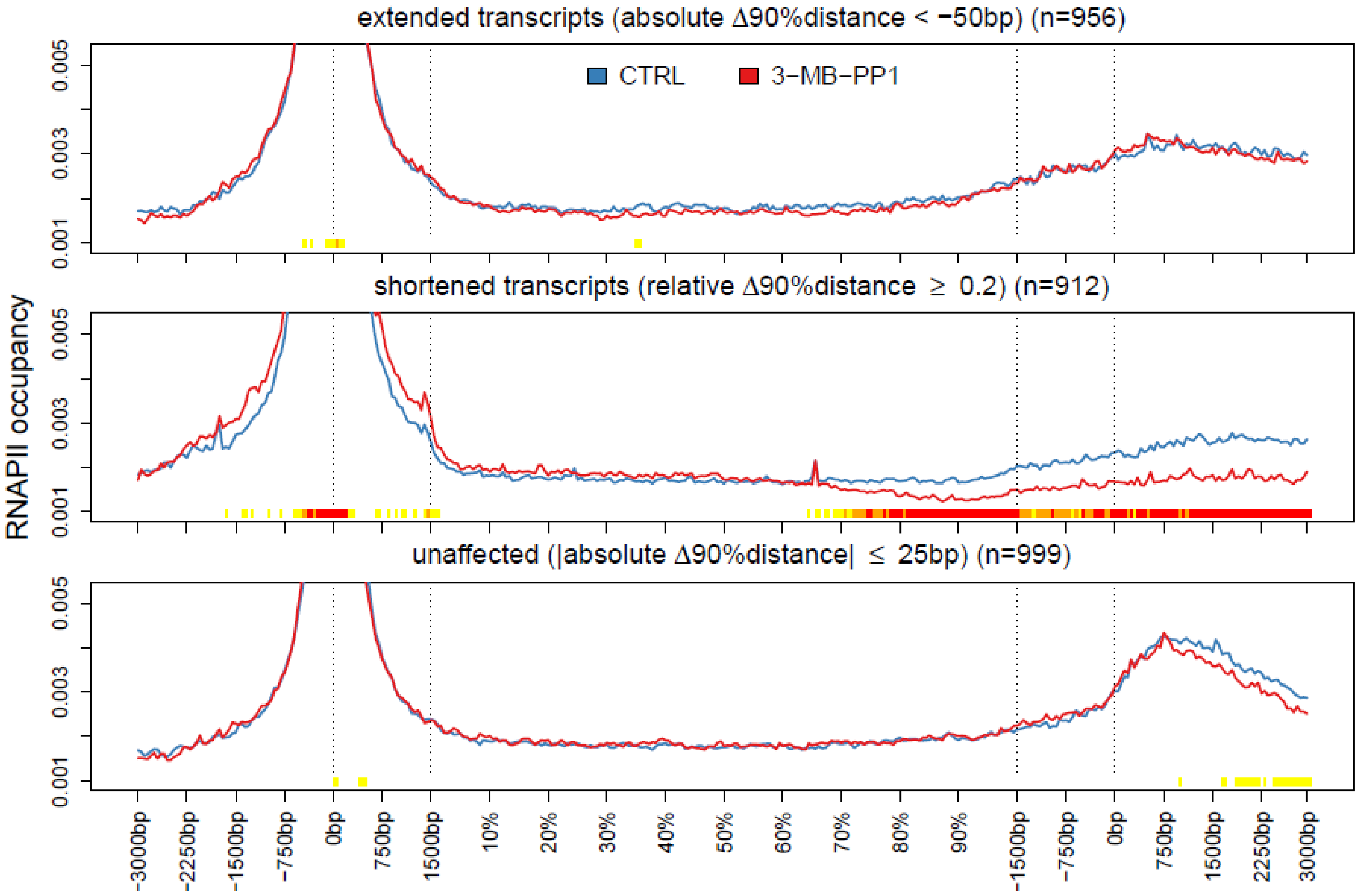

Appendix Fig. S14

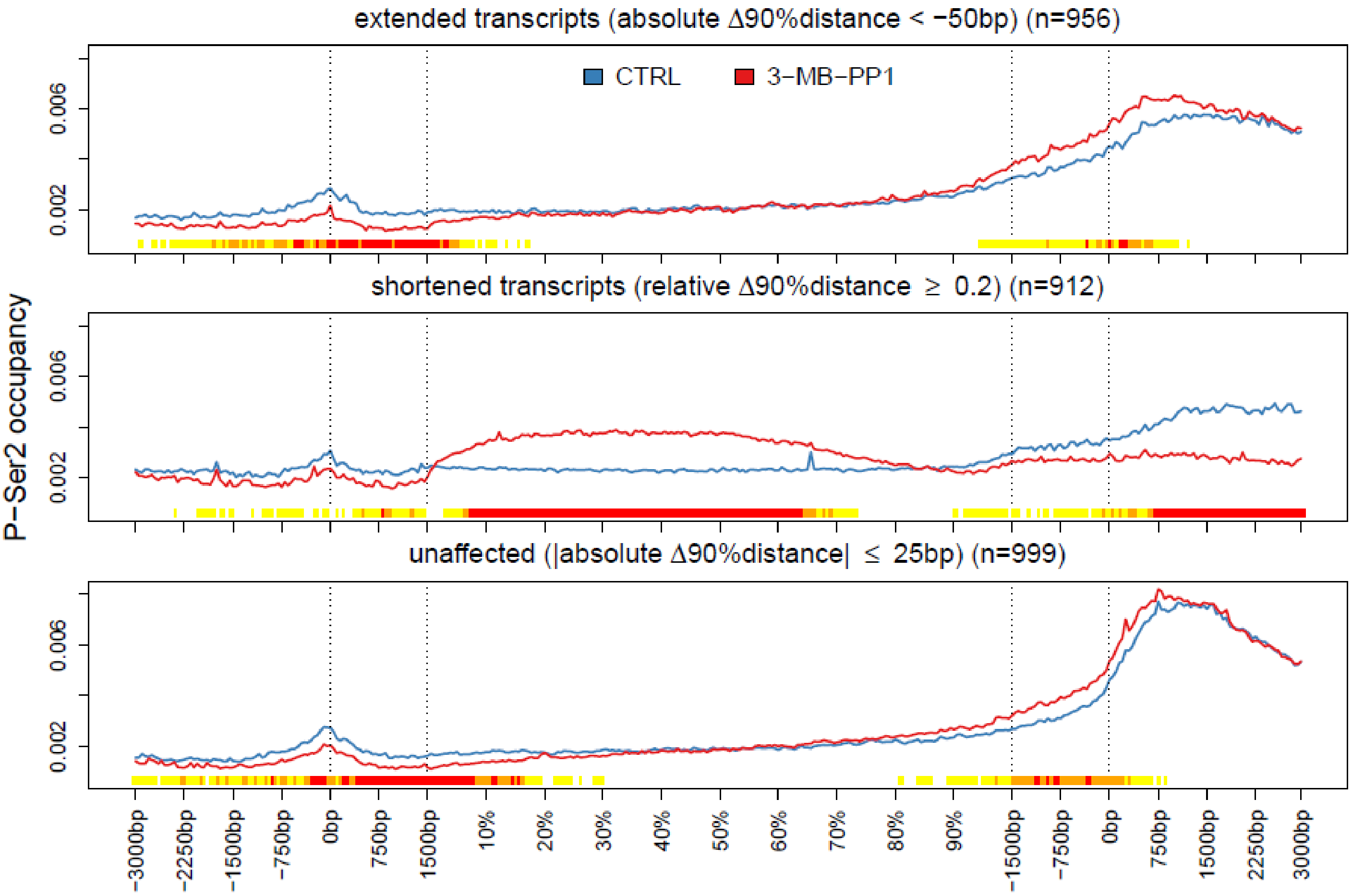

Appendix Fig. S15

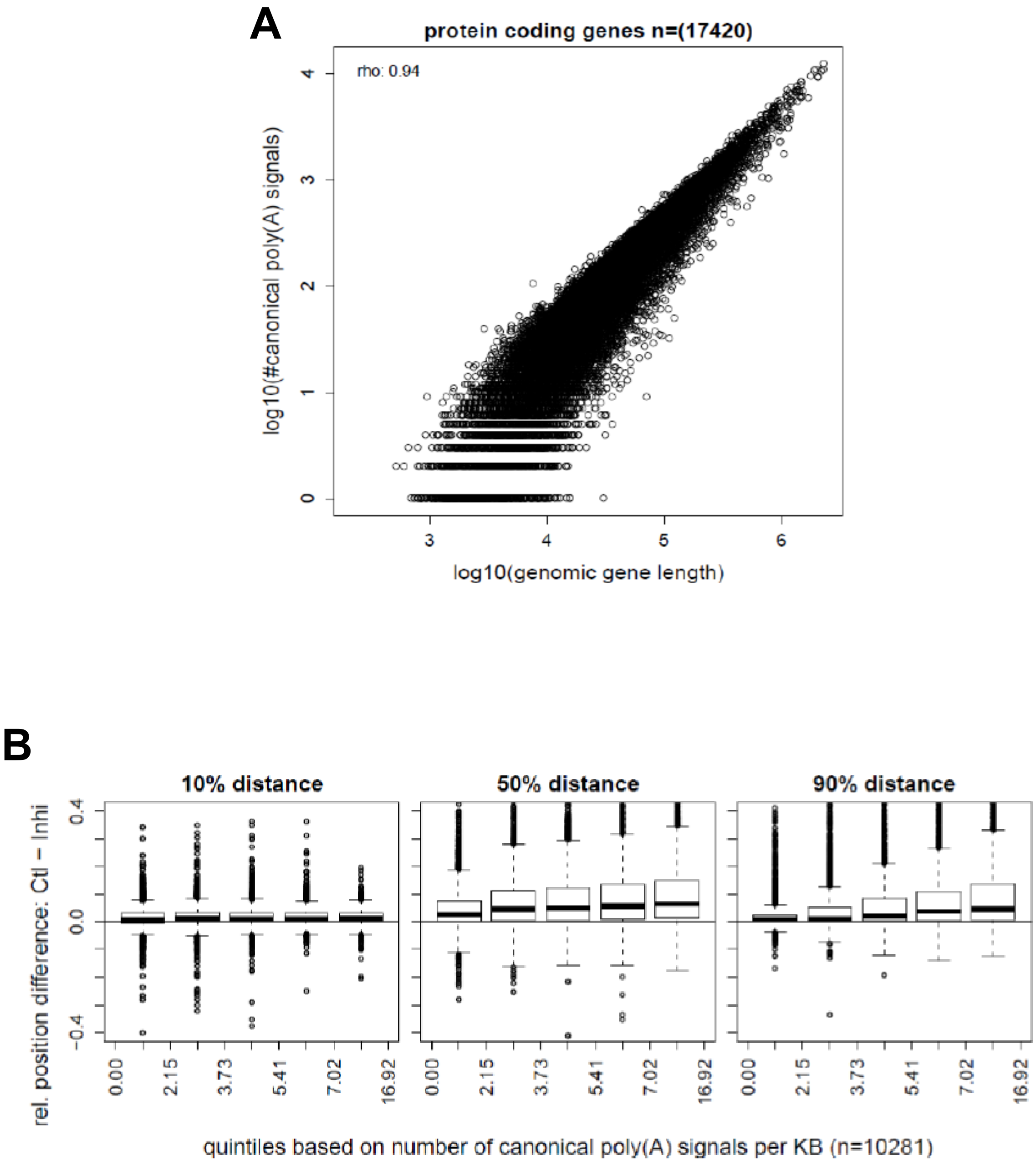

Supplement: Supplementary file 1 — Appendix [file EMBR-20-e47592-s001.pdf]
