# Supplementary material for: CDK12 controls G1/S progression by regulating RNAPII processivity at core DNA replication genes
Source: EMBO Rep. 2019 Jul 25;20(9):e47592. doi: 10.15252/embr.201847592 (PMC6727028; doi:10.15252/embr.201847592)
Supplement: Supplementary file 2 — Expanded View Figures PDF [file EMBR-20-e47592-s002.pdf]

Expanded View Figures

Figure EV1. Preparation and characterization of AS CDK12 HCT116 cell line.

A Depiction of *CDK12* locus, genome editing, and genotyping strategy. Schema of *CDK12* locus, with exon numbers shown above the *CDK12* gene depiction (top). Primers used for genotyping PCR surrounding exon 6 of *CDK12* gene are shown as horizontal arrows, PCR product is depicted as full horizontal line, and *BslI* restriction sites are indicated by vertical arrows. *BslI* restriction site created by genome editing is shown in green. Size (bp) of genotyping PCR product and *BslI* restriction fragments are indicated (middle). DNA subjected to genome editing and corresponding protein sequences in exon 6 of *CDK12* genes are shown; the underlined DNA sequence in WT *CDK12* allele underwent genome editing to create silent mutation preventing alternative splicing (nucleotide in blue), *BslI* restriction site, and to convert F813 to G813 (nucleotides in red) in AS CDK12. Engineered G813 in AS CDK12 is indicated in red (bottom).

B Characterization of AS CDK12 clone by a AS primer-specific PCR. Exon 6 in *CDK12* gene is shown as a black box. Edited DNA in the AS CDK12 is marked by a red vertical line in the exon 6. Genotyping primers specific for WT (black arrows) and AS CDK12 (red arrow) are shown, and genotyping PCR product is depicted by a dashed line with size (in bp) indicated above (top). Ethidium bromide-stained agarose gel visualizing 352 bp PCR product from PCR mixture using either WT- (left) or AS-specific (right) forward primer (bottom).

C CCNK/CDK12 complex shows comparable properties in the AS and WT CDK12 HCT116 cell lines. Western blot analysis of protein levels (input) and association [determined by immunoprecipitation (IP)] of CCNK and CDK12 in the indicated cell lines. No Ab corresponds to a control immunoprecipitation without antibody. A representative image of three replicates is shown.

D Quantification of individual P-Ser modifications in the CTD of RNAPII after CDK12 inhibition. Amounts of individual proteins and CTD modifications presented in Fig 1D and in another two biological replicates from short film exposures were quantified by ImageJ software. All protein levels were normalized to a corresponding tubulin loading control, and samples without treatment in each time point (CTRL) were considered as 1; $n = 3$ biological replicates and error bars are standard error of the mean (SEM).

Source data are available online for this figure.

 © 2019 The Authors

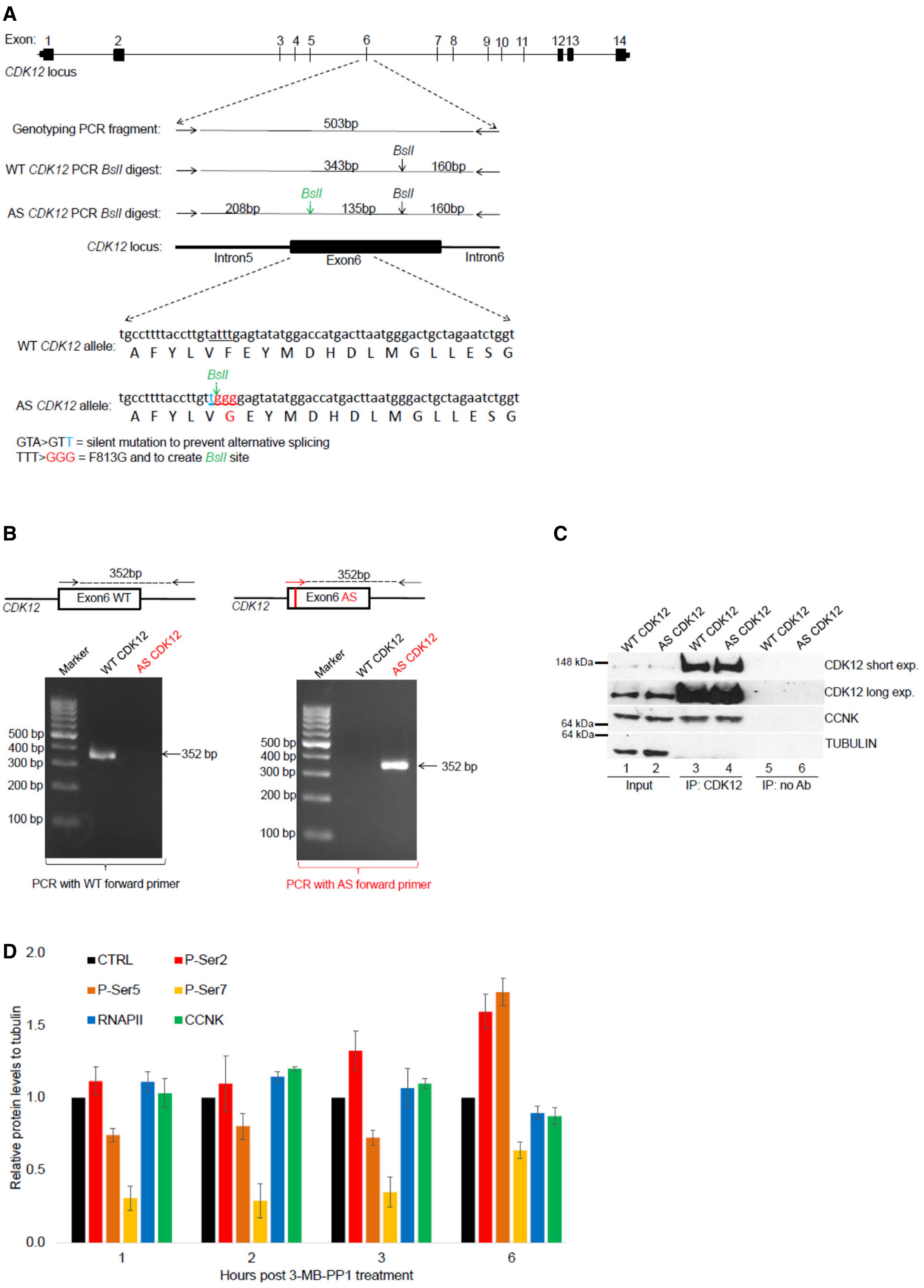
A
Exon: 1 2 3 4 5 6 7 8 9 10 11 12 13 14
CDK12 locus
Genotyping PCR fragment: 503bp
WT CDK12 PCR BslI digest: 343bp BslI 160bp
AS CDK12 PCR BslI digest: 208bp BslI 135bp BslI 160bp
CDK12 locus: Intron5 Exon6 Intron6
WT CDK12 allele: tgccttttaccttgtatttgagtatatggaccatgacttaatgggactgctagaatctggt
A F Y L V F E Y M D H D L M G L L E S G
BslI
AS CDK12 allele: tgccttttaccttgttgggagtatatggaccatgacttaatgggactgctagaatctggt
A F Y L V G E Y M D H D L M G L L E S G
GTA>GTT = silent mutation to prevent alternative splicing
TTT>GGG = F813G and to create BslI site
B
352bp
CDK12 Exon6 WT
CDK12 Exon6 AS
Marker WT CDK12 AS CDK12
500 bp 400 bp 300 bp 200 bp 100 bp
352 bp
PCR with WT forward primer
PCR with AS forward primer
C
WT CDK12 AS CDK12 WT CDK12 AS CDK12 WT CDK12 AS CDK12
148 kDa
64 kDa
64 kDa
CDK12 short exp.
CDK12 long exp.
CCNK
TUBULIN
1 2 3 4 5 6
Input IP: CDK12 IP: no Ab
D
CTRL P-Ser2 P-Ser5 P-Ser7 RNAPII CCNK
Relative protein levels to tubulin
0.0 0.5 1.0 1.5 2.0
1 2 3 6
Hours post 3-MB-PP1 treatment

Figure EV1.

Figure EV2. CDK12 kinase activity is essential for optimal G1/S progression.

A 3-MB-PP1 does not affect cell cycle progression in WT HCT116 cells. The experiment was performed as shown in Fig 2A. *n* = 3; representative result is shown.

B THZ531 causes G1/S progression defect in WT HCT116 cells arrested by serum starvation. Flow cytometry profiles of control (−THZ531) or 350 nM THZ531(+THZ531)-treated cells from the experiment outlined in Fig 2A. Red arrow points to the onset of the G1/S progression defect in THZ531-treated cells. *n* = 3 replicates; representative result is shown.

C CDK12 inhibition delays G1/S progression in thymidine/nocodazole-arrested AS CDK12 HeLa cells. Flow cytometry profiles of control (−3-MB-PP1) or 3-MB-PP1 (+3-MB-PP1) treated cells from the experiment shown in Fig 2A. Red arrow points to the onset of the G1/S progression defect in 3-MB-PP1-treated cells. *n* = 3 replicates; representative result is shown.

D Experimental outline. AS CDK12 HCT116 cells were arrested by serum starvation for 72 h and released into the serum-containing medium with (+) or without (−) 3-MB-PP1. 3-MB-PP1 was washed away and replaced with fresh medium at indicated times after the release, and all samples were subjected to flow cytometry analyses at 15 h after the release.

E G1/S progression delay can be rescued by removal of CDK12 inhibitor at early G1 phase. Flow cytometry profiles of propidium iodide-labeled cells from the experiment depicted in Fig EV2D. CTRL = control samples without the 3-MB-PP1. *n* = 3 replicates; representative result is shown.

© 2019 The Authors

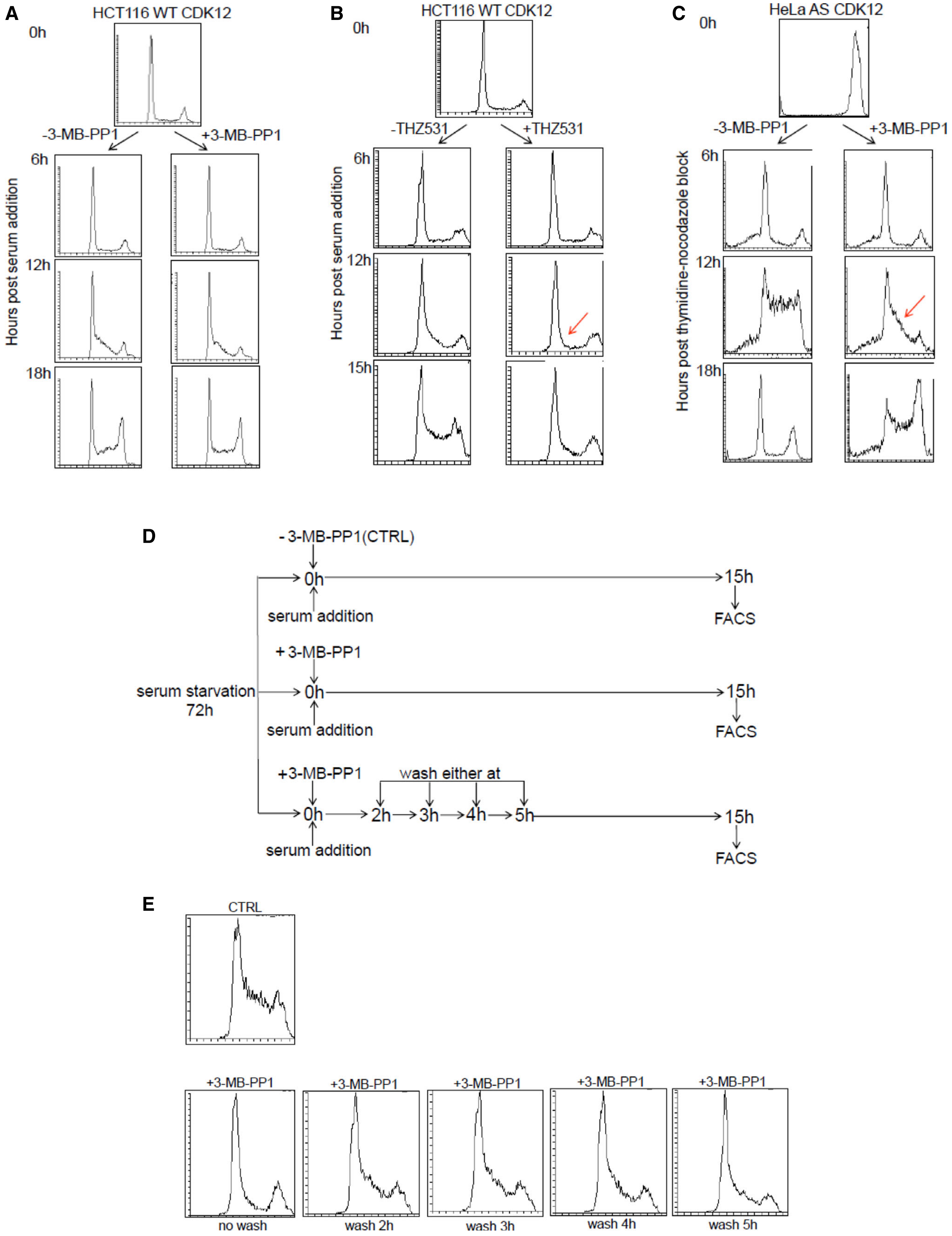
A
HCT116 WT CDK12
0h
-3-MB-PP1
+3-MB-PP1
6h
12h
18h
Hours post serum addition
B
HCT116 WT CDK12
0h
-THZ531
+THZ531
6h
12h
15h
Hours post serum addition
C
HeLa AS CDK12
0h
-3-MB-PP1
+3-MB-PP1
6h
12h
18h
Hours post thymidine-nocodazole block
D
- 3-MB-PP1(CTRL)
0h
15h
serum addition
FACS
+ 3-MB-PP1
serum starvation 72h
0h
15h
serum addition
FACS
+3-MB-PP1
wash either at
0h
2h
3h
4h
5h
15h
serum addition
FACS
E
CTRL
+3-MB-PP1
no wash
wash 2h
wash 3h
wash 4h
wash 5h

Figure EV2.

Figure EV3. CDK12 catalytic activity controls expression of core DNA replication genes.

A CDK12 inhibition down-regulates DNA replication-related genes. GSEA analysis based on log2 fold-changes in 3′end RNA-seq data upon CDK12 inhibition. Normalized enrichment scores (NES) are shown for significant GO terms (FDR q-val < 0.05) with negative NES, i.e., associated with down-regulation. Functions related to DNA replication are marked by the red rectangles.

B Expression of crucial DNA replication genes is dependent on the CDK12 kinase activity. Comparison of log2 fold-changes versus log2 mean expression in 3′end RNA-seq data and depicts down-regulated DNA replication genes (−0.85 > log2 fold-change, $P < 0.01$) after 5-h CDK12 inhibition.

C Validation of 3′end RNA-seq for select non-regulated genes by RT–qPCR. See Fig 3D for legend. $n = 3$ replicates, error bars represent SEM.

D Inhibition of CDK12 kinase does not affect mRNA degradation of select DNA repair and replication transcripts. AS CDK12 HCT116 cells were treated with ActD (1 μg/ml) either in the presence (red line) or absence (CTRL) (blue line) of 3-MB-PP1. Total mRNA was isolated at indicated time points, and levels of indicated mRNAs normalized to *HPRT1* were measured by RT–qPCR. Graphs present mRNA levels relative to untreated cells (time 0 h set to 1). $n = 3$ independent experiments, error bars are SEM.

E Expression of core DNA replication proteins is dependent on the CDK12 kinase activity. See legend in Fig 3E.

F, G CCNK depletion diminishes mRNA and protein expression of DNA replication genes. RT–qPCR of mRNA levels (F) and Western blot of protein levels (G) in AS CDK12 HCT116 cells treated with control (CTRL) or CCNK siRNAs for 36 h. mRNA levels were normalized to *GAPDH* mRNA expression. $n = 3$ replicates for RT–qPCR (F), error bars indicate SEM. In (G), a representative experiment from three replicates is shown.

Source data are available online for this figure.

© 2019 The Authors

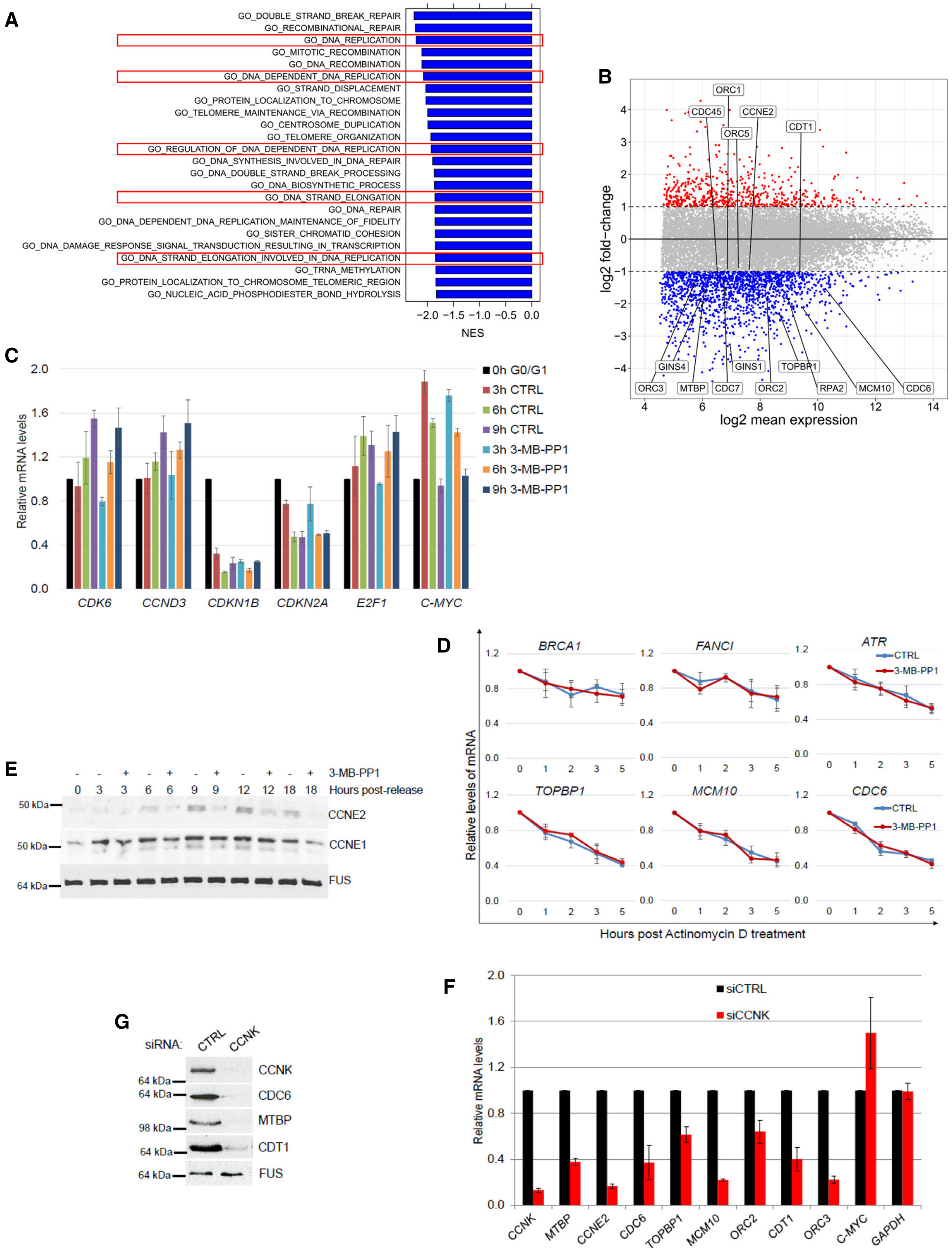
A
GO_DOUBLE_STRAND_BREAK_REPAIR
GO_RECOMBINATIONAL_REPAIR
GO_DNA_REPLICATION
GO_MITOTIC_RECOMBINATION
GO_DNA_RECOMBINATION
GO_DNA_DEPENDENT_DNA_REPLICATION
GO_STRAND_DISPLACEMENT
GO_PROTEIN_LOCALIZATION_TO_CHROMOSOME
GO_TELOMERE_MAINTENANCE_VIA_RECOMBINATION
GO_CENTROSOME_DUPLICATION
GO_TELOMERE_ORGANIZATION
GO_REGULATION_OF_DNA_DEPENDENT_DNA_REPLICATION
GO_DNA_SYNTHESIS_INVOLVED_IN_DNA_REPAIR
GO_DNA_DOUBLE_STRAND_BREAK_PROCESSING
GO_DNA_BIOSYNTHETIC_PROCESS
GO_DNA_STRAND_ELONGATION
GO_DNA_REPAIR
GO_DNA_DEPENDENT_DNA_REPLICATION_MAINTENANCE_OF_FIDELITY
GO_SISTER_CHROMATID_COHESION
GO_DNA_DAMAGE_RESPONSE_SIGNAL_TRANSDUCTION_RESULTING_IN_TRANSCRIPTION
GO_DNA_STRAND_ELONGATION_INVOLVED_IN_DNA_REPLICATION
GO_TRNA_METHYLATION
GO_PROTEIN_LOCALIZATION_TO_CHROMOSOME_TELOMERIC_REGION
GO_NUCLEIC_ACID_PHOSPHODIESTER_BOND_HYDROLYSIS
−2.0 −1.5 −1.0 −0.5 0.0
NES
B
ORC1
CDC45
CCNE2
ORC5
CDT1
GINS4
GINS1
TOPBP1
ORC3
MTBP
CDC7
ORC2
RPA2
MCM10
CDC6
log2 fold−change
log2 mean expression
C
0h G0/G1
3h CTRL
6h CTRL
9h CTRL
3h 3-MB-PP1
6h 3-MB-PP1
9h 3-MB-PP1
Relative mRNA levels
CDK6
CCND3
CDKN1B
CDKN2A
E2F1
C-MYC
D
BRCA1
FANCI
ATR
CTRL
3-MB-PP1
TOPBP1
MCM10
CDC6
Relative levels of mRNA
Hours post Actinomycin D treatment
E
3-MB-PP1
Hours post-release
0 3 3 6 6 9 9 12 12 18 18
50 kDa
CCNE2
CCNE1
64 kDa
FUS
F
siCTRL
siCCNK
Relative mRNA levels
CCNK
MTBP
CCNE2
CDC6
TOPBP1
MCM10
ORC2
CDT1
ORC3
C-MYC
GAPDH
G
siRNA:
CTRL
CCNK
64 kDa
98 kDa
CDC6
MTBP
CDT1
FUS

Figure EV3.

Figure EV4. CDK12 directs expression of replication and DNA damage response genes downstream of the E2F/RB pathway.

A CDK12 directs expression of DNA replication genes downstream of the E2F/RB pathway. Graphs present ChIP-qPCR data for E2F1 and E2F3 in AS CDK12 HCT116 cells either treated or not with 3-MB-PP1 for 4 h. qPCR primers were designed at promoters of indicated genes. $n = 3$ replicates; error bars represent SEM. Ir is intergenic region; noAb corresponds to no antibody immunoprecipitation control.

B CDK12 inhibition does not lead to differential recruitment of RNAPII to E2F target genes. The plots show log2 fold-changes of RNAPII occupancy on promoters of E2F target genes (*y*-axis) plotted against corresponding log2 fold-changes in mRNA expression from nuclear RNA-seq (*x*-axis). Promoter occupancy was quantified as read counts in the ±3 kb regions around the transcription start site (TSS). For each gene, we selected the transcript with the most read counts in the RNAPII ChIP-seq samples (normalized to library size) in the ±3 kb regions around the TSS and transcription termination site (TTS). Corresponding RNAPII ChIP-seq and nuclear RNA-seq experiments are presented in Fig 5A and B. E2F target genes were obtained from Bracken *et al* [40]; rho = Spearman rank correlation coefficient.

© 2019 The Authors

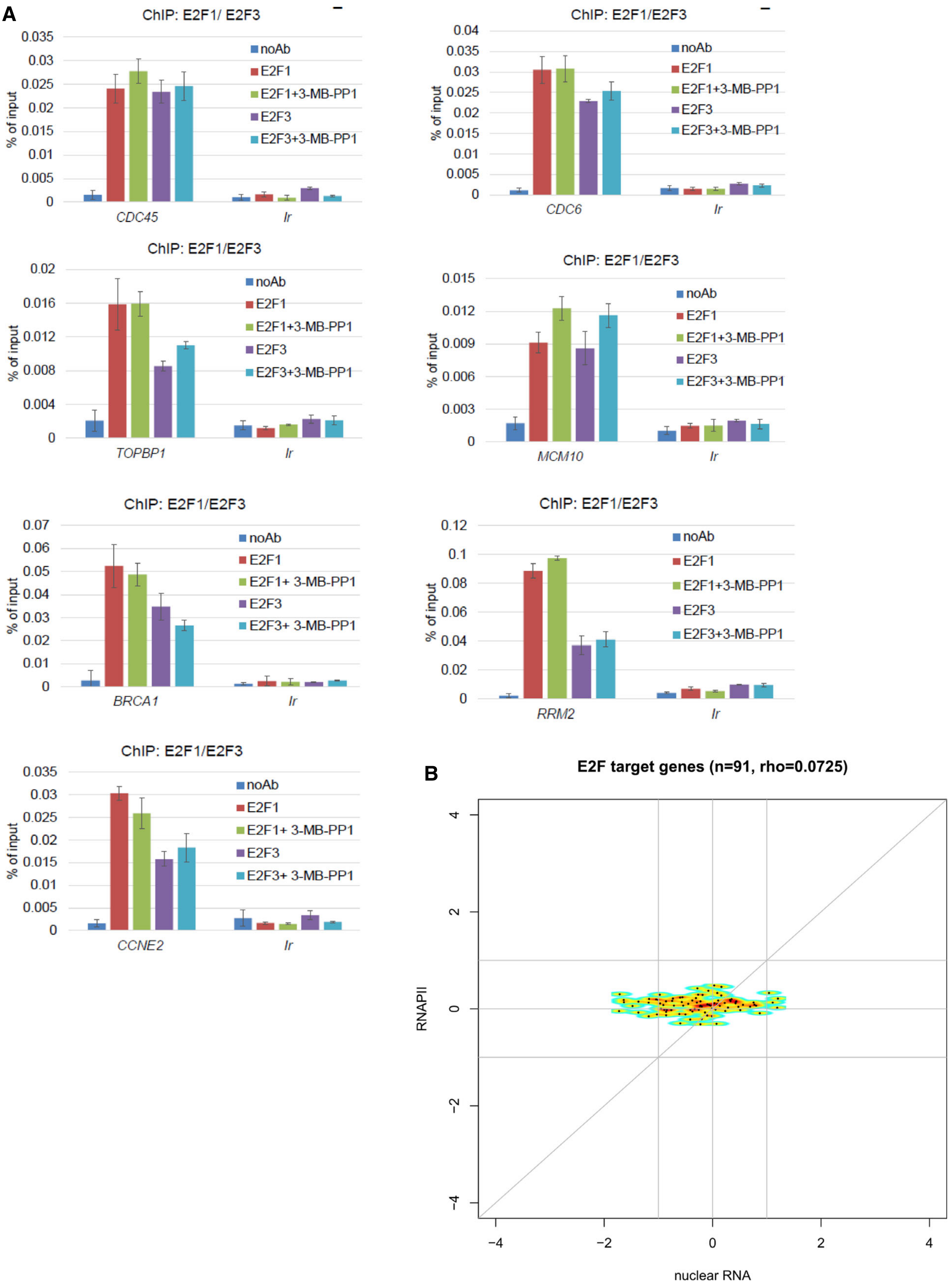
A
ChIP: E2F1/ E2F3
ChIP: E2F1/E2F3
% of input
noAb
E2F1
E2F1+3-MB-PP1
E2F3
E2F3+3-MB-PP1
E2F1+ 3-MB-PP1
E2F3+ 3-MB-PP1
CDC45
CDC6
TOPBP1
MCM10
BRCA1
RRM2
CCNE2
Ir
B
E2F target genes (n=91, rho=0.0725)
RNAPII
nuclear RNA

Figure EV4.

Figure EV5. Inhibition of CDK12 leads to diminished RNAPII processivity on down-regulated genes.

A High correlation between gene expression changes in nuclear and 3′end RNA-seq data. Graph compares log2 fold-changes in nuclear and 3′end RNA-seq data determined with DESeq2. rho = Spearman rank correlation coefficient.

B Inhibition of CDK12 affects the expression of similar subsets of genes in nuclear and 3′end RNA-seq data. See Fig 5A for legend. Venn diagrams are shown for significantly down-regulated (log2 fold-change < 0, $P \leq 0.01$) and up-regulated (log2 fold-change > 0, $P \leq 0.01$) genes.

C P-Ser5 occupancy shows shifts after CDK12 inhibition. Metagene analysis of P-Ser5 ChIP-seq data as described in Fig 5B and C.

D SPT6 shows diminished relative occupancy at 3′ends of down-regulated genes upon CDK12 inhibition. Metagene analysis of SPT6 ChIP-seq data as described in Fig 5B and C.

E CDK12 inhibition does not affect SPT6/RNAPII association in cells. Western blot analyses of SPT6 and RNAPII interaction after 4-h treatment with the 3-MB-PP1 in AS CDK12 HCT116 cells. Representative image from three replicates is shown.

Source data are available online for this figure.

© 2019 The Authors

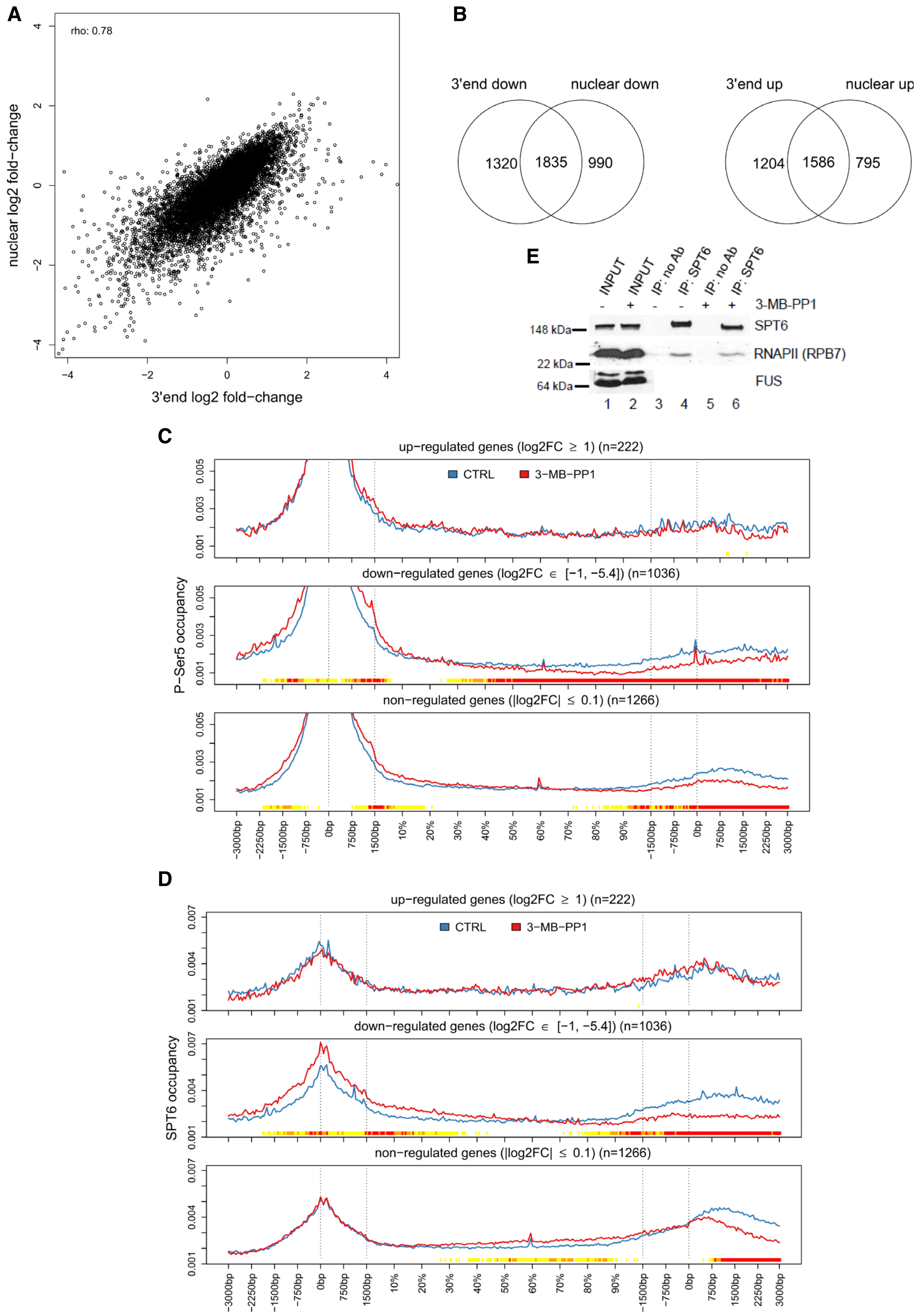
A
rho: 0.78
nuclear log2 fold−change
3'end log2 fold−change
B
3'end down
nuclear down
1320
1835
990
3'end up
nuclear up
1204
1586
795
E
INPUT
INPUT
IP: no Ab
IP: SPT6
IP: no Ab
IP: SPT6
3-MB-PP1
148 kDa
SPT6
22 kDa
RNAPII (RPB7)
64 kDa
FUS
1 2 3 4 5 6
C
up−regulated genes (log2FC ≥ 1) (n=222)
CTRL
3−MB−PP1
down−regulated genes (log2FC ∈ [−1, −5.4]) (n=1036)
non−regulated genes (|log2FC| ≤ 0.1) (n=1266)
P−Ser5 occupancy
D
up−regulated genes (log2FC ≥ 1) (n=222)
CTRL
3−MB−PP1
down−regulated genes (log2FC ∈ [−1, −5.4]) (n=1036)
non−regulated genes (|log2FC| ≤ 0.1) (n=1266)
SPT6 occupancy

Figure EV5.
