# Supplementary figures and images for: CDK12 controls G1/S progression by regulating RNAPII processivity at core DNA replication genes
Source: EMBO Rep. 2019 Jul 25;20(9):e47592. doi: 10.15252/embr.201847592 (PMC6727028; doi:10.15252/embr.201847592)

## Expanded View Figure: 1c

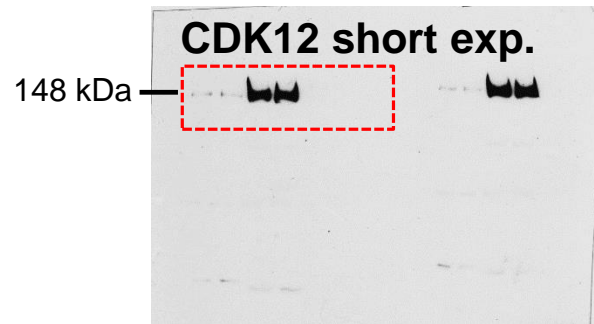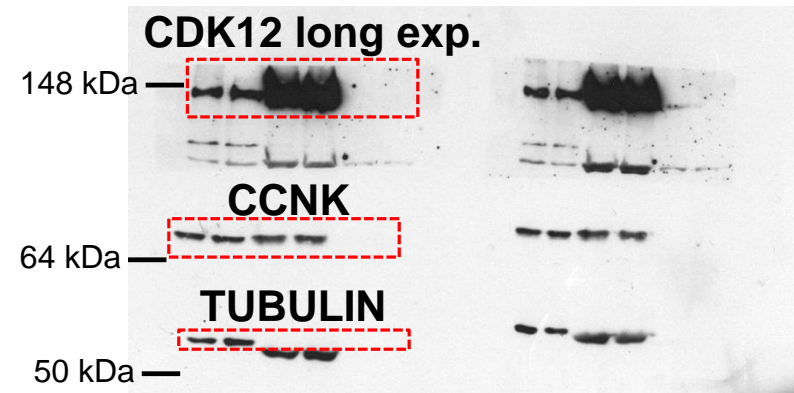

Supplement: Supplementary file 6 — Source Data for Expanded View [file EMBR-20-e47592-s010.zip › Source_Data_for_EV_Figures/Source_Data_for_FigEV1.pdf]

## Expanded View Figure: 3e

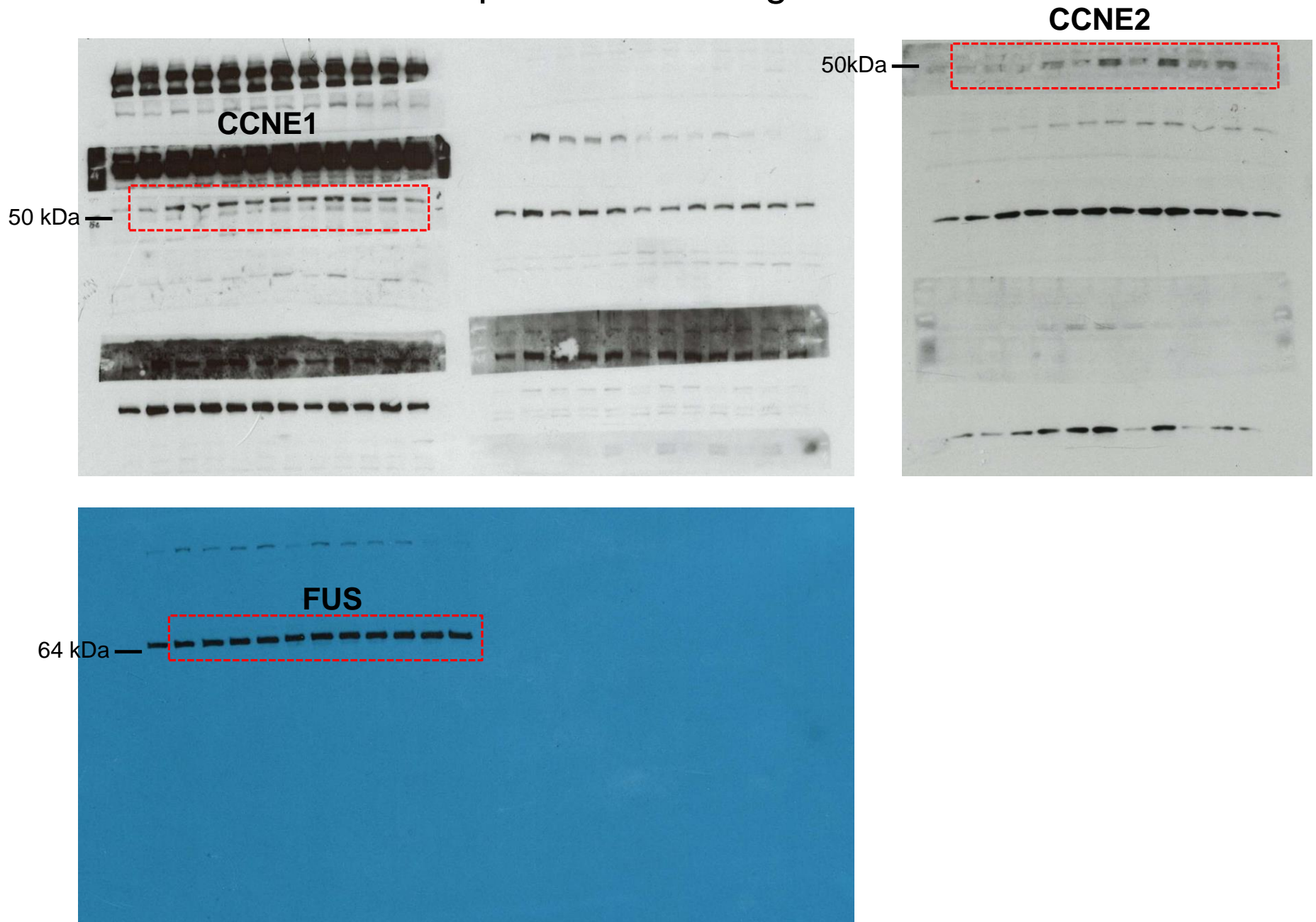

# Expanded View Figure: 3g

## CDC6

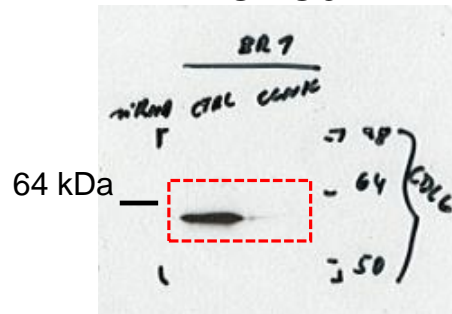

## FUS

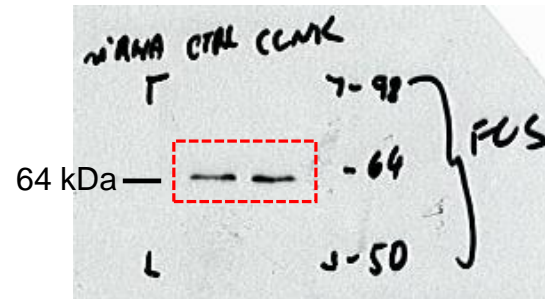

## CCNK

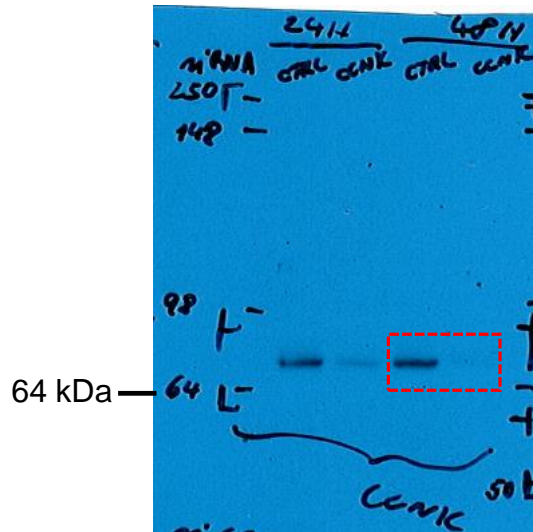

## MTBP

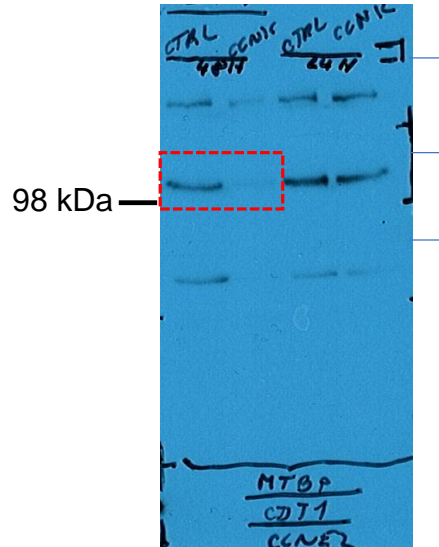

## CDT1

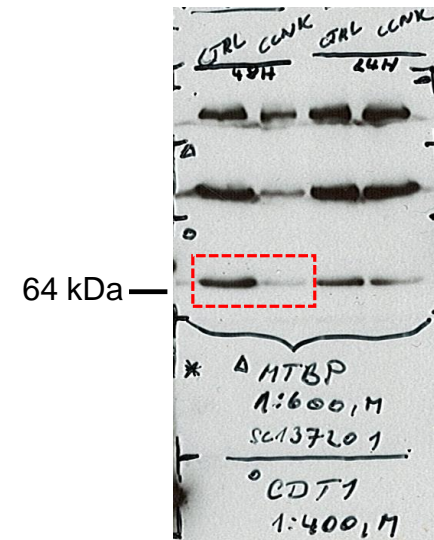

Supplement: Supplementary file 6 — Source Data for Expanded View [file EMBR-20-e47592-s010.zip › Source_Data_for_EV_Figures/Source_Data_for_FigEV3.pdf]

## Expanded View Figure: 5e

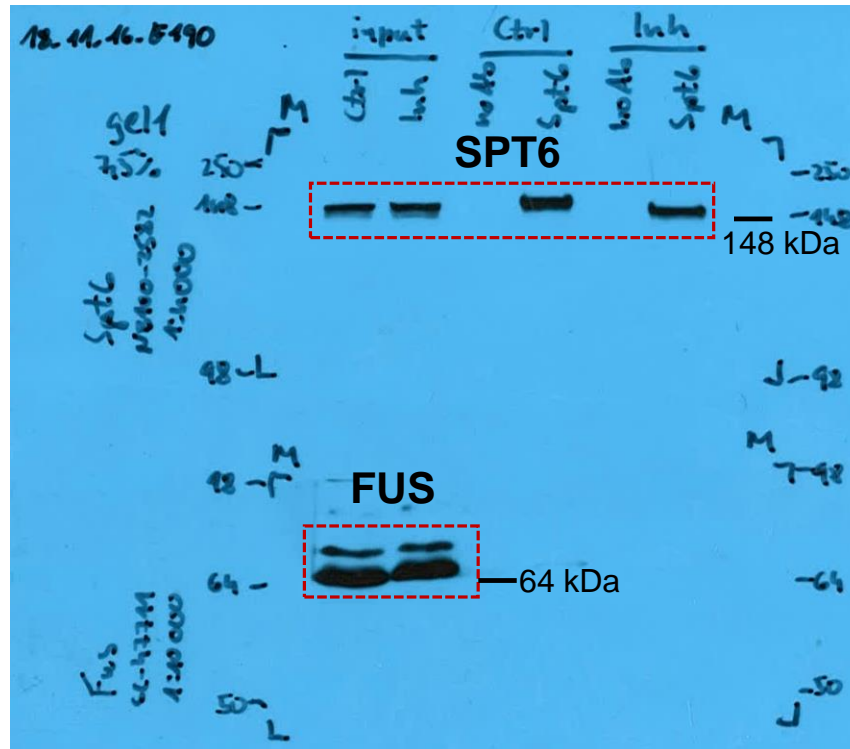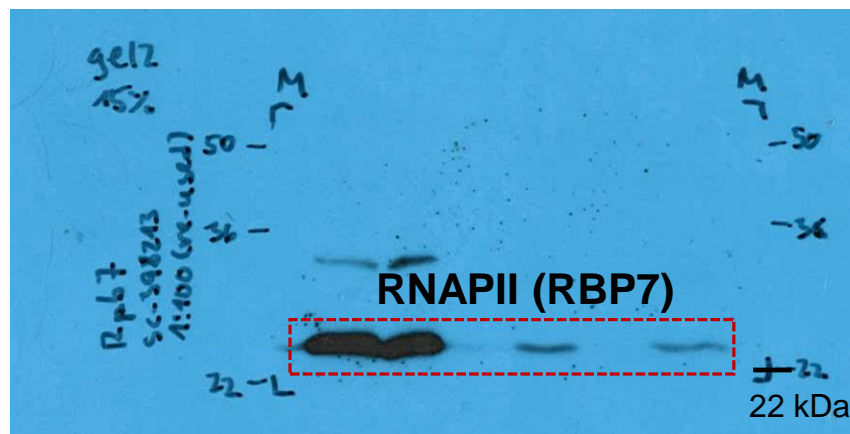

Supplement: Supplementary file 6 — Source Data for Expanded View [file EMBR-20-e47592-s010.zip › Source_Data_for_EV_Figures/Source_Data_for_FigEV5.pdf]

Figure: 1d

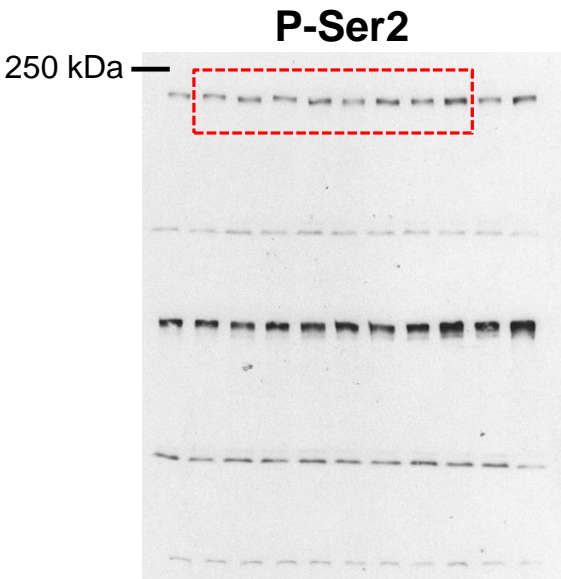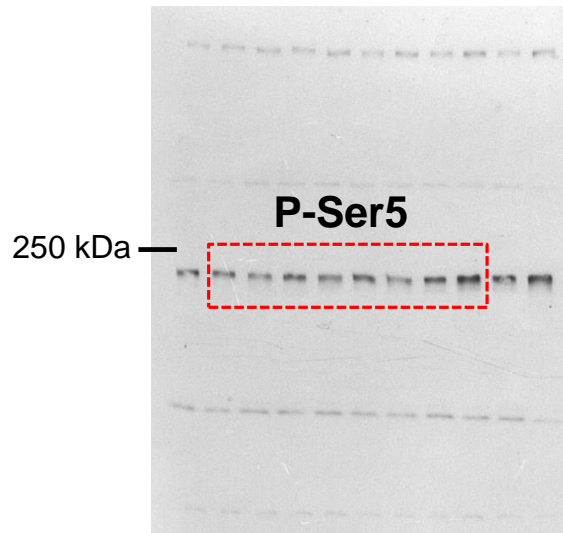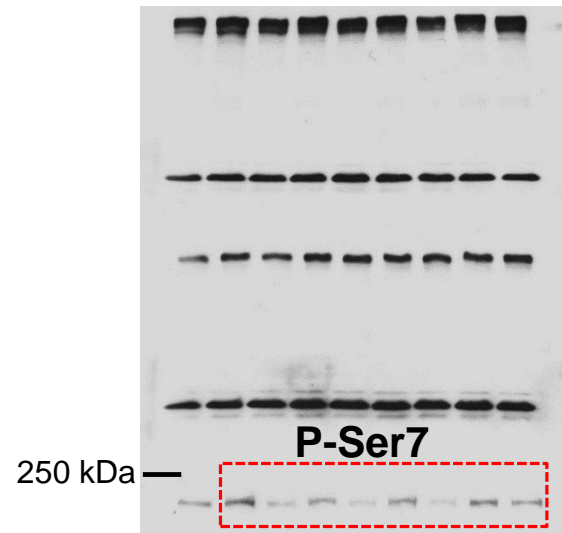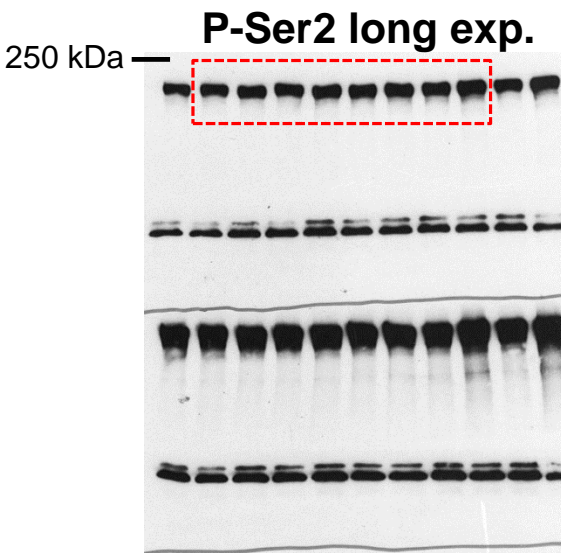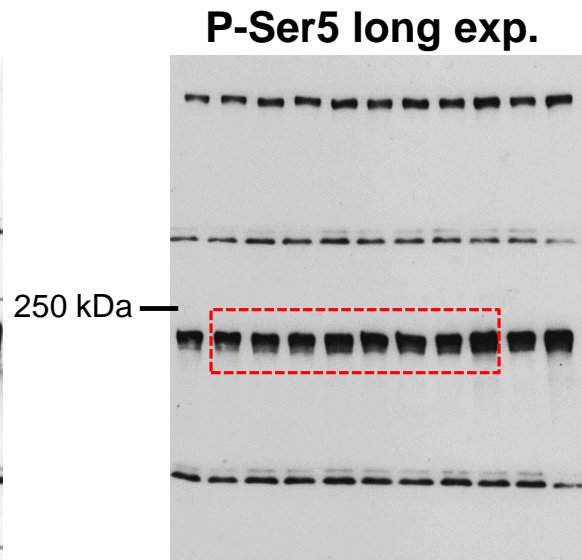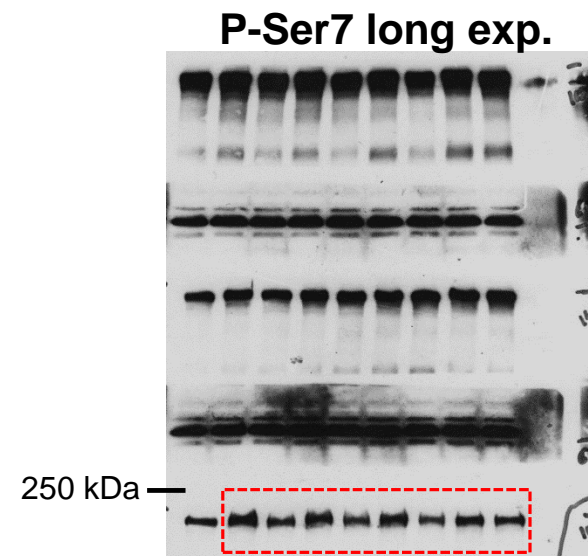

Figure: 1d

**RNAPII (RPB1)**

250 kDa

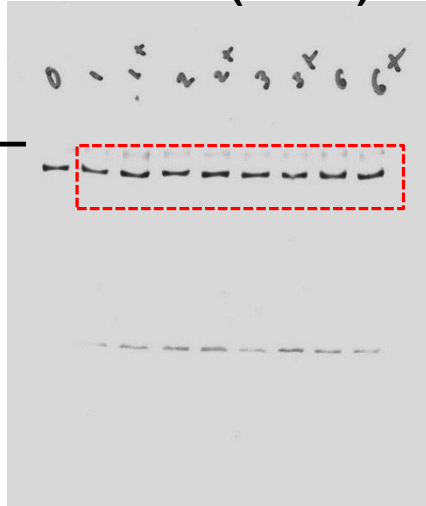

**CCNK**

64 kDa

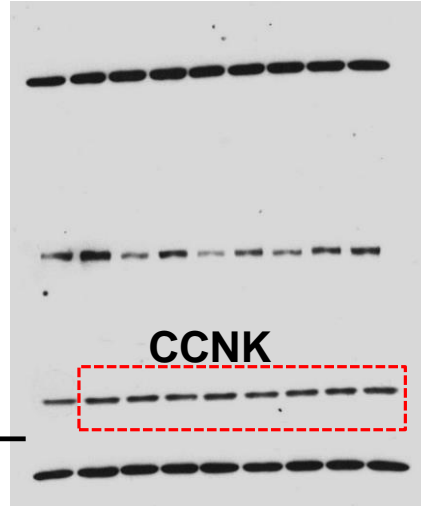

**FUS**

64 kDa

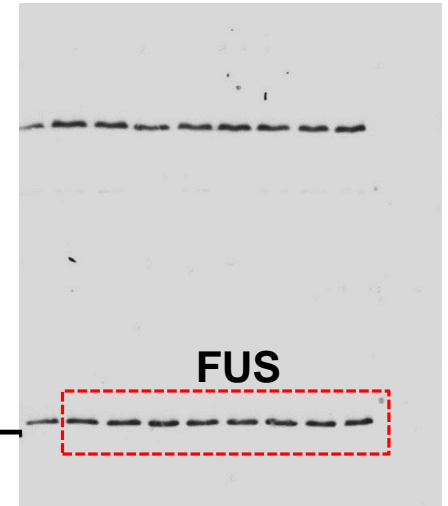

**TUBULIN**

50 kDa

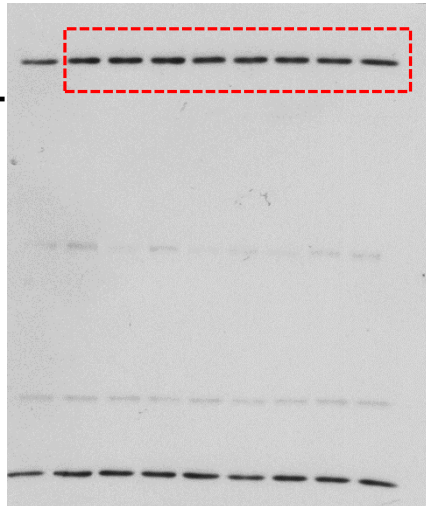

Supplement: Supplementary file 8 — Source Data for Figure 1 [file EMBR-20-e47592-s006.pdf]

Figure: 2d

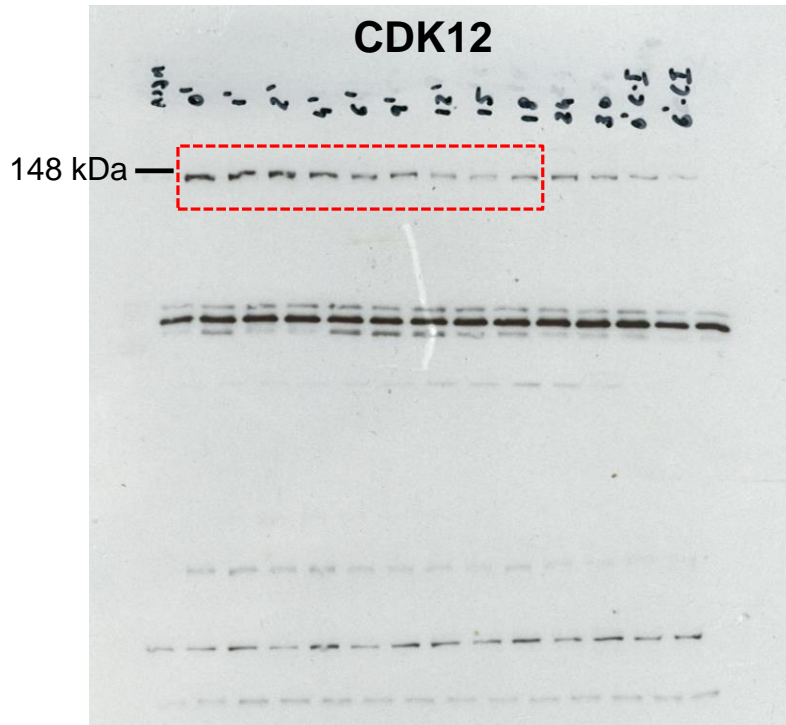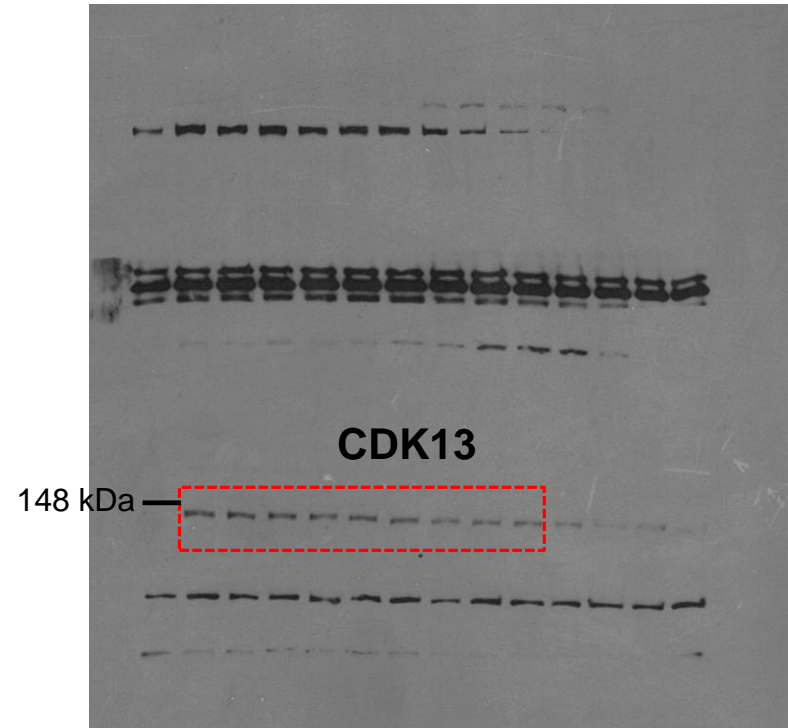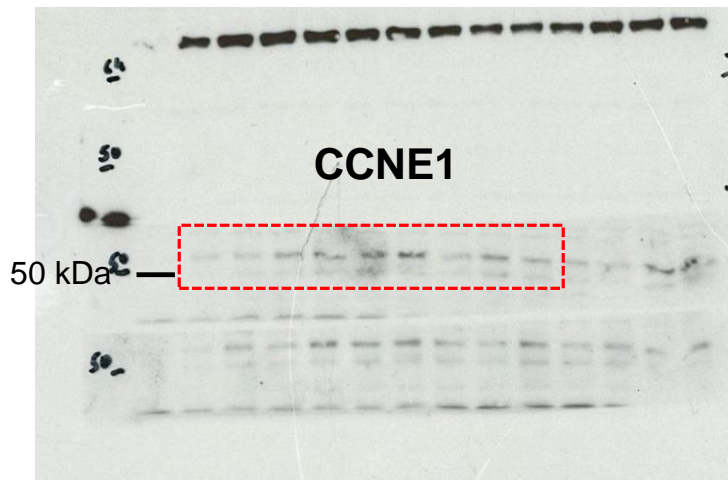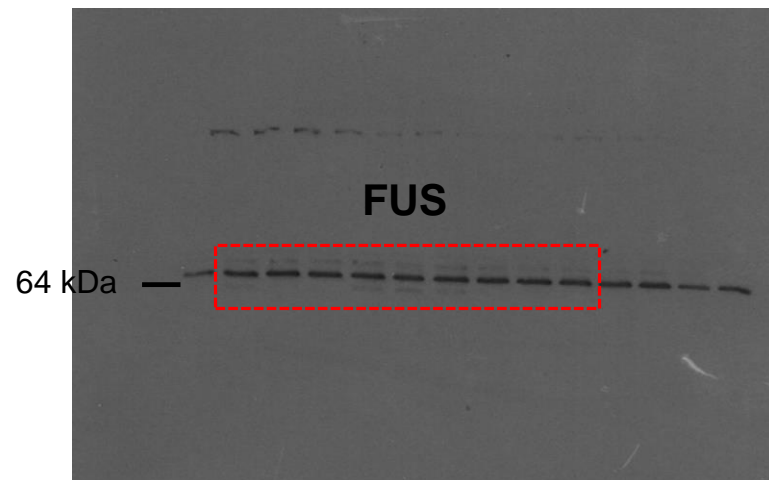

Figure: 2d

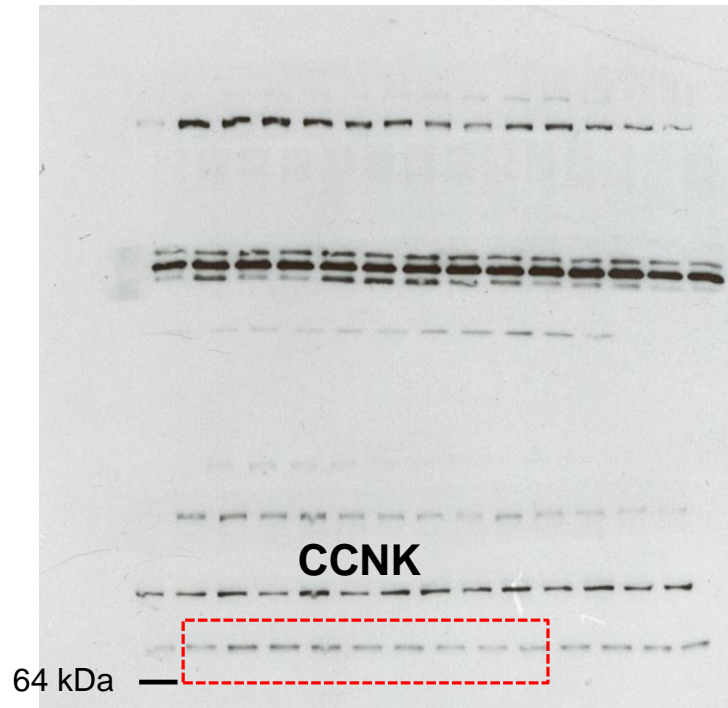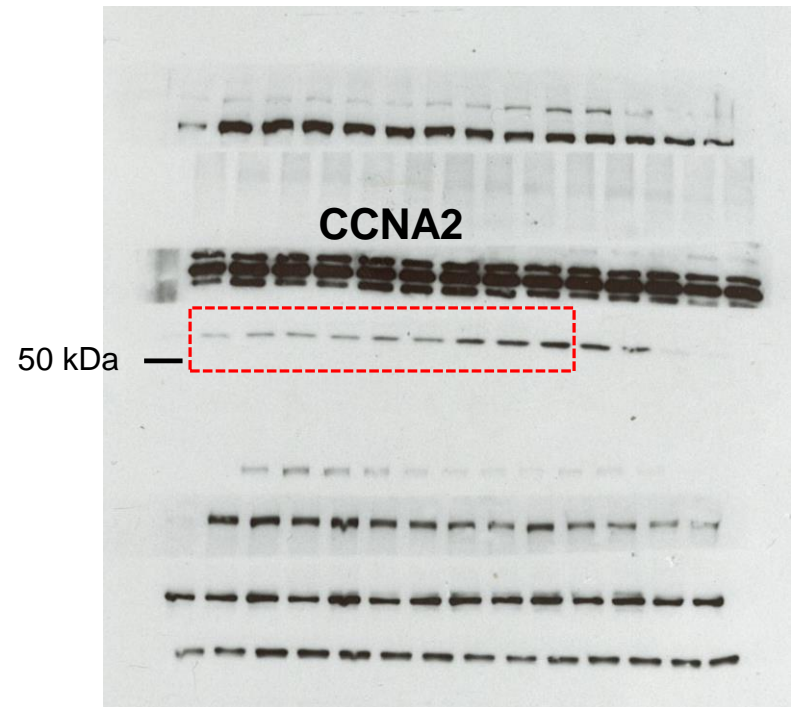

Figure: 2h

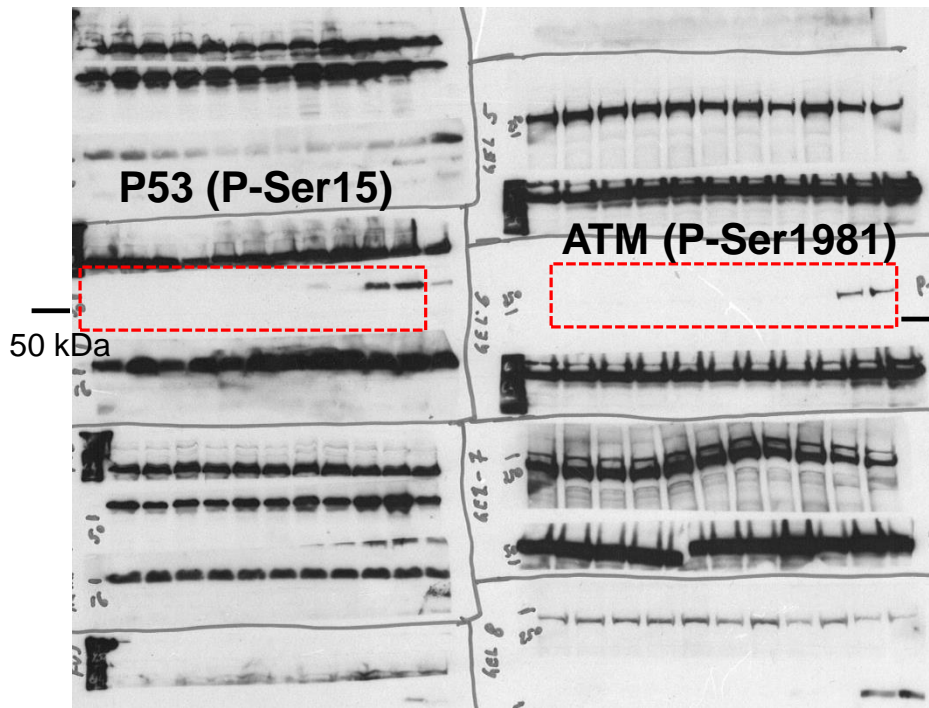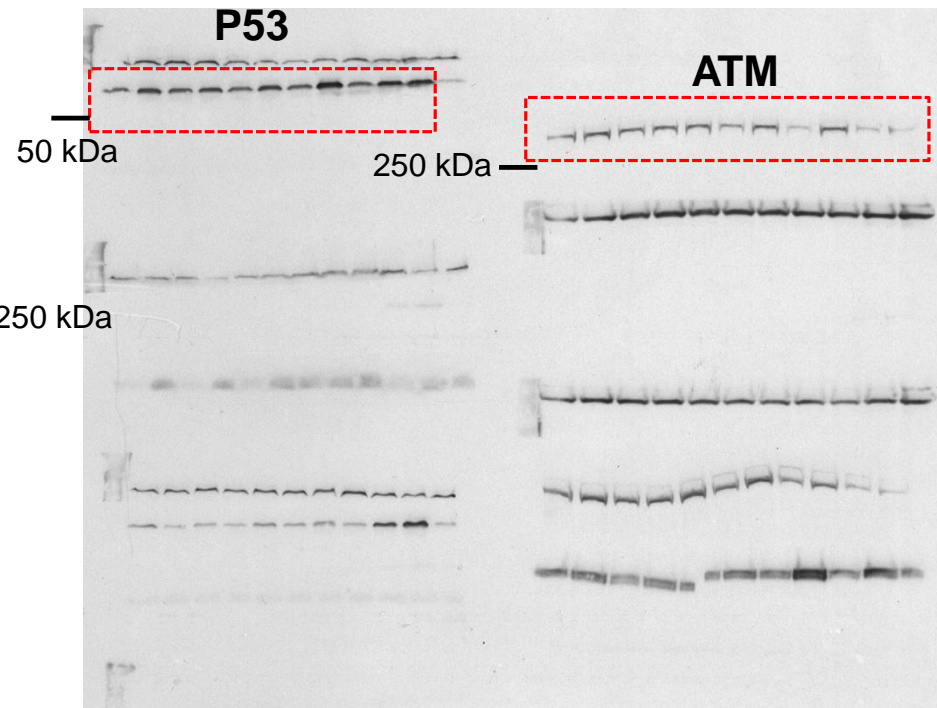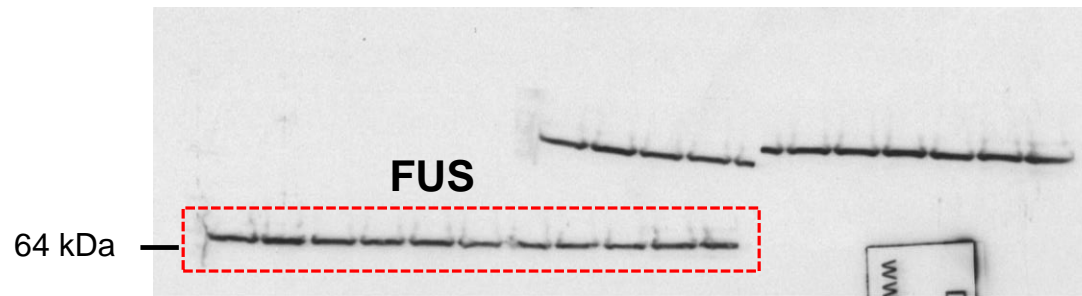

Supplement: Supplementary file 9 — Source Data for Figure 2 [file EMBR-20-e47592-s007.pdf]

Figure: 3e

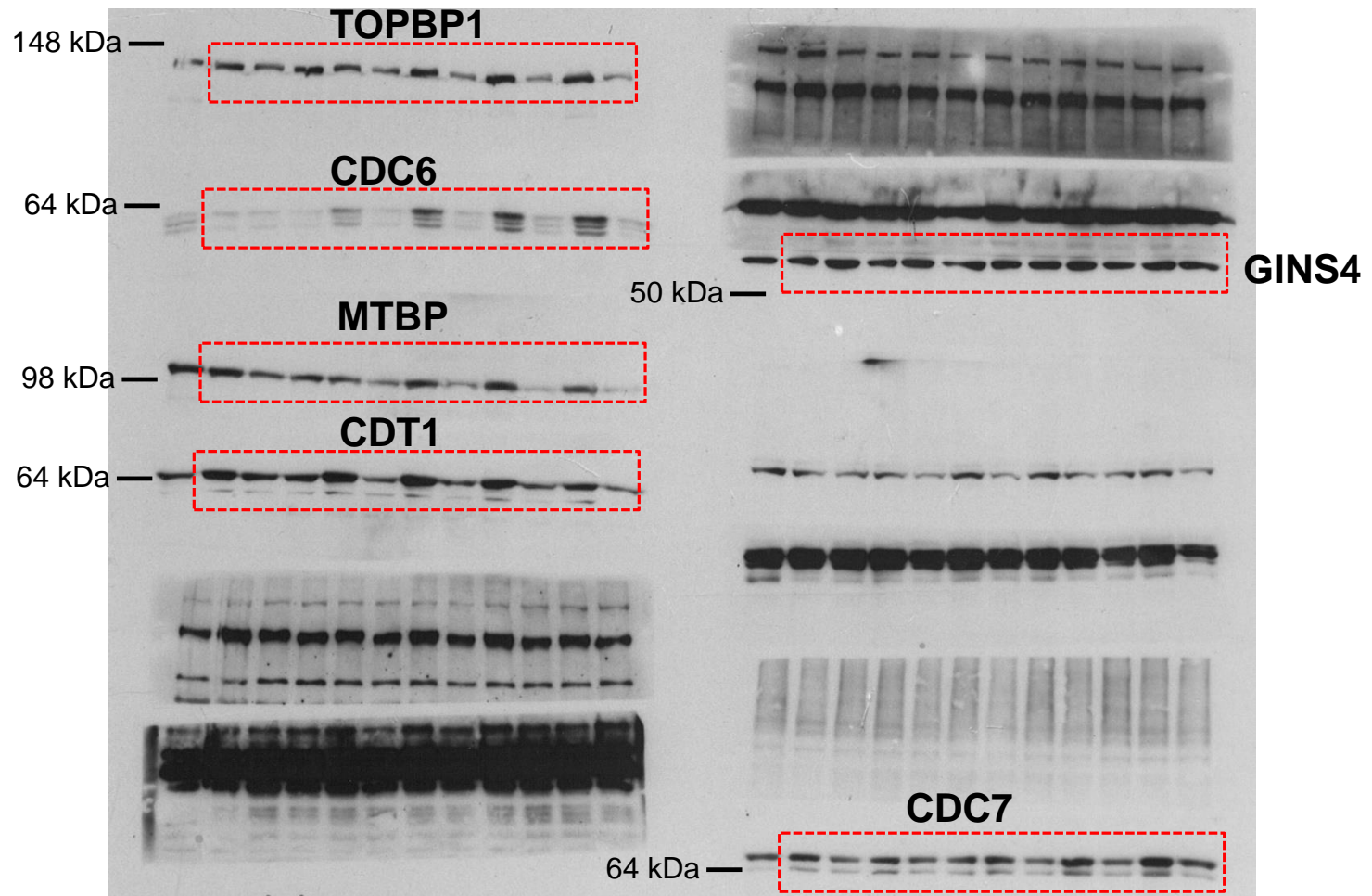

Figure: 3e

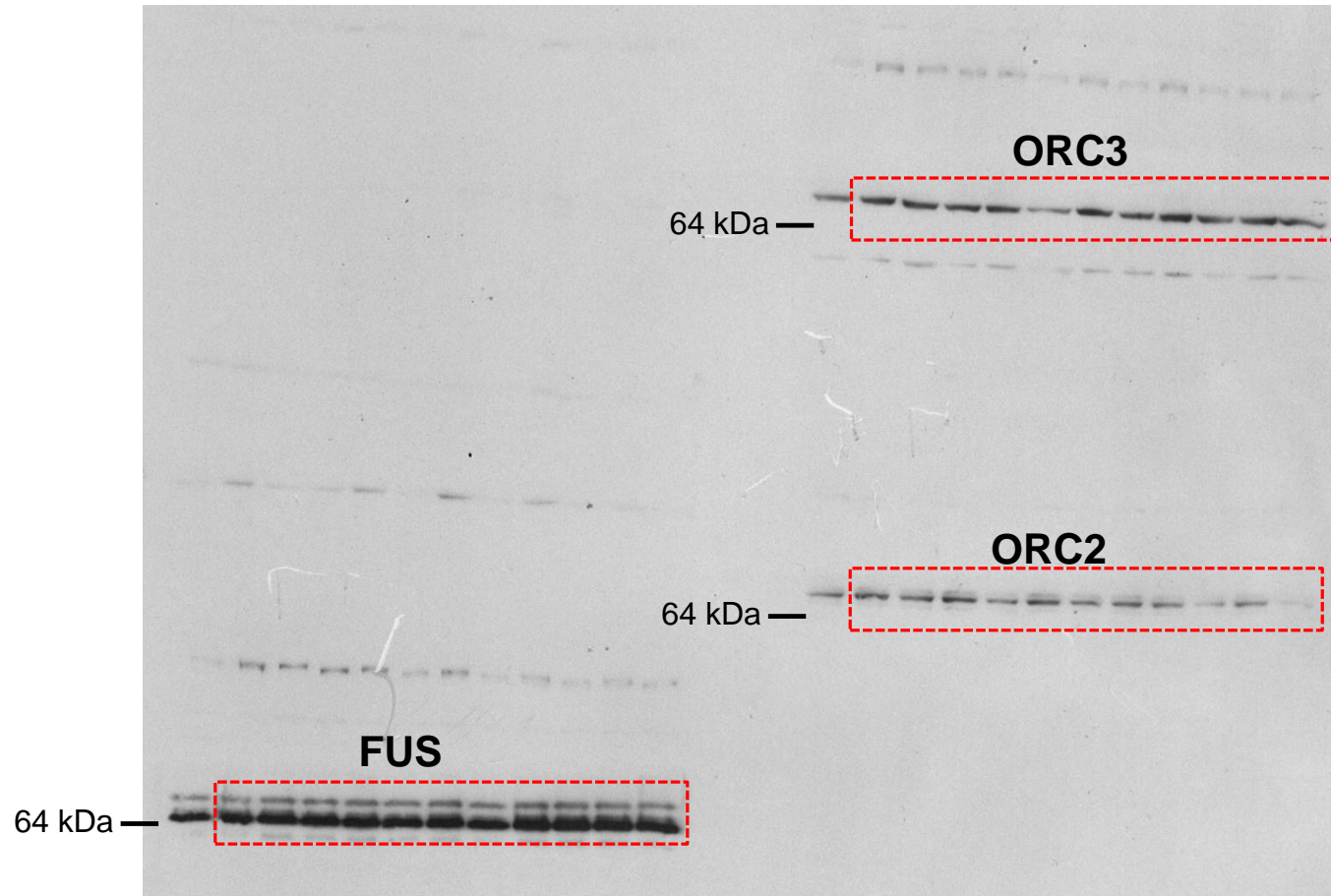

Figure: 3f

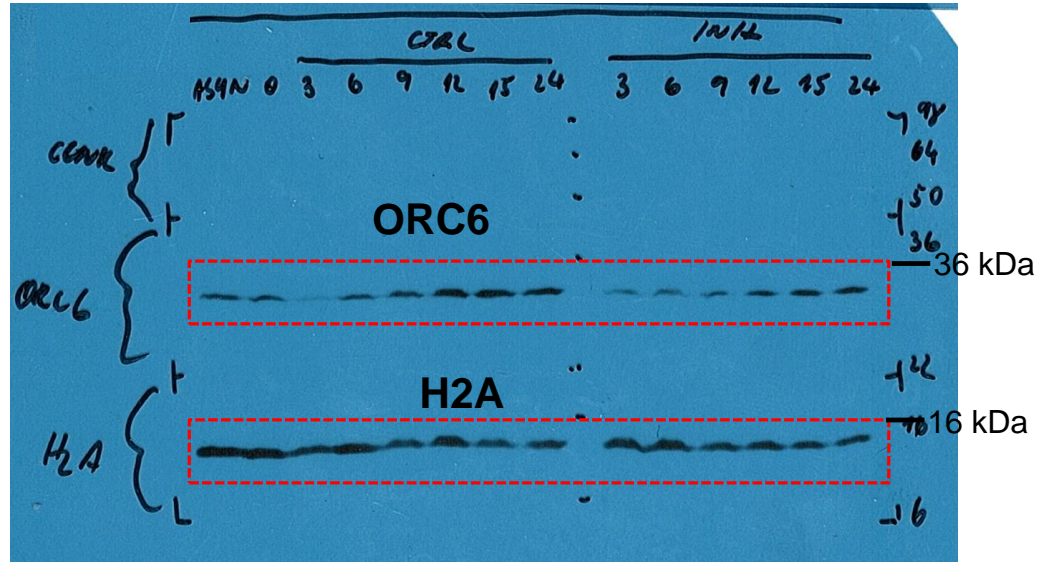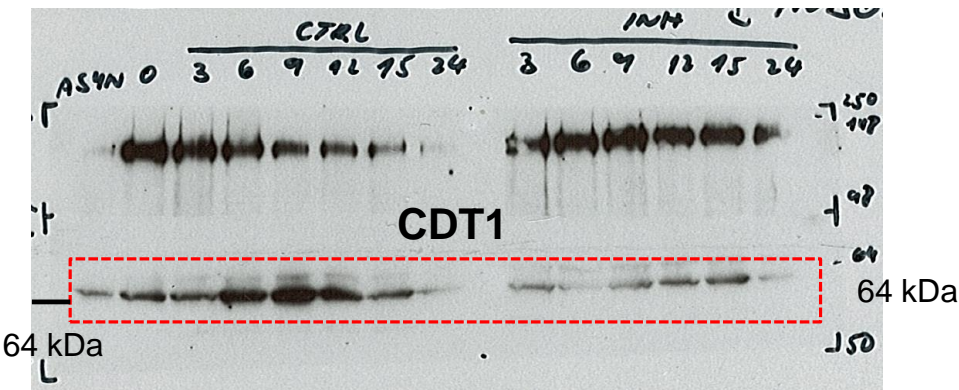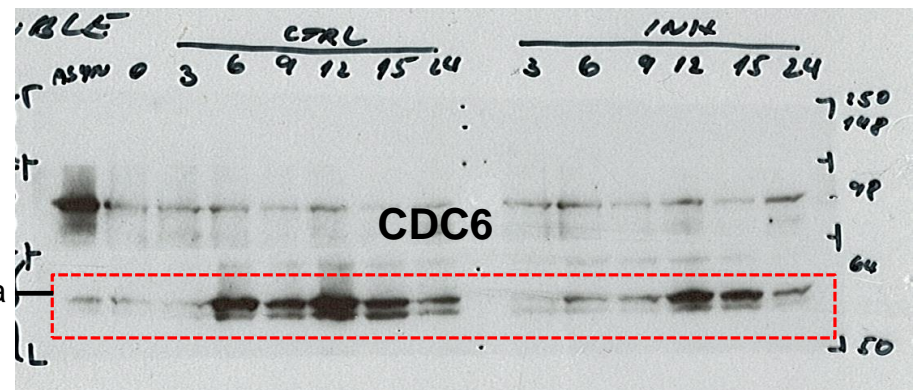

Supplement: Supplementary file 10 — Source Data for Figure 3 [file EMBR-20-e47592-s008.pdf]

Figure: 4c

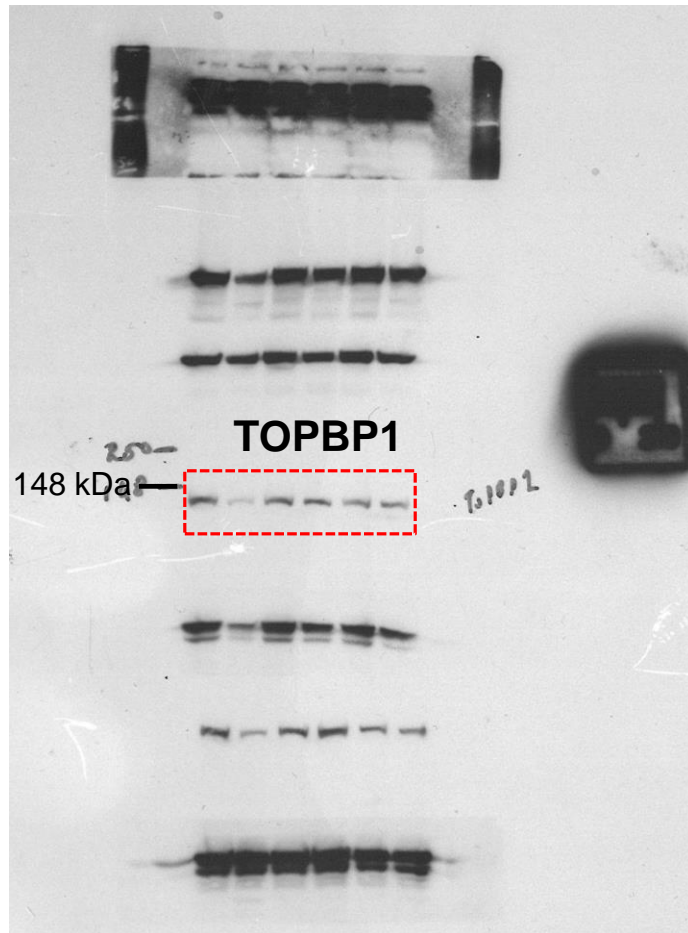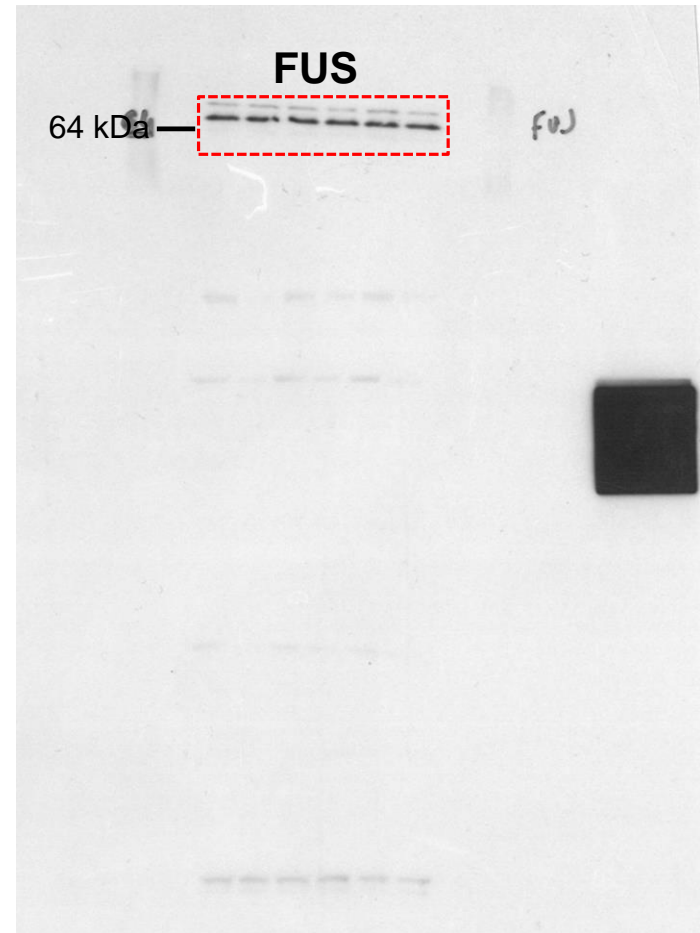

Figure: 4e

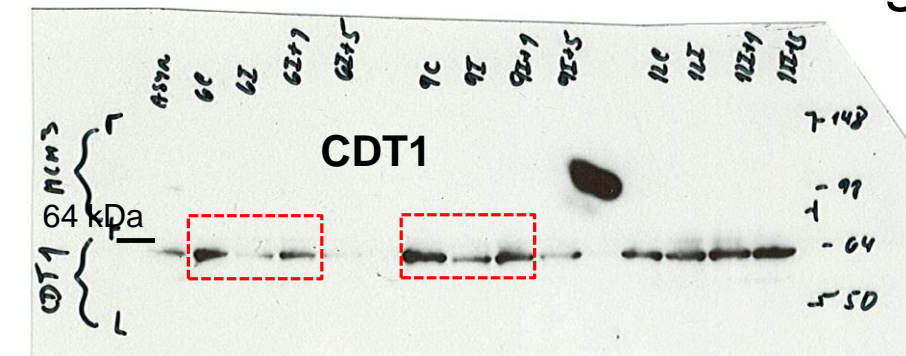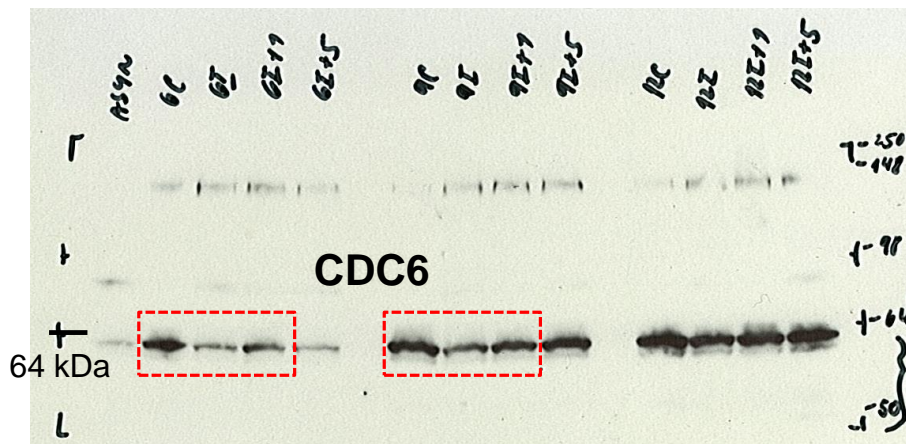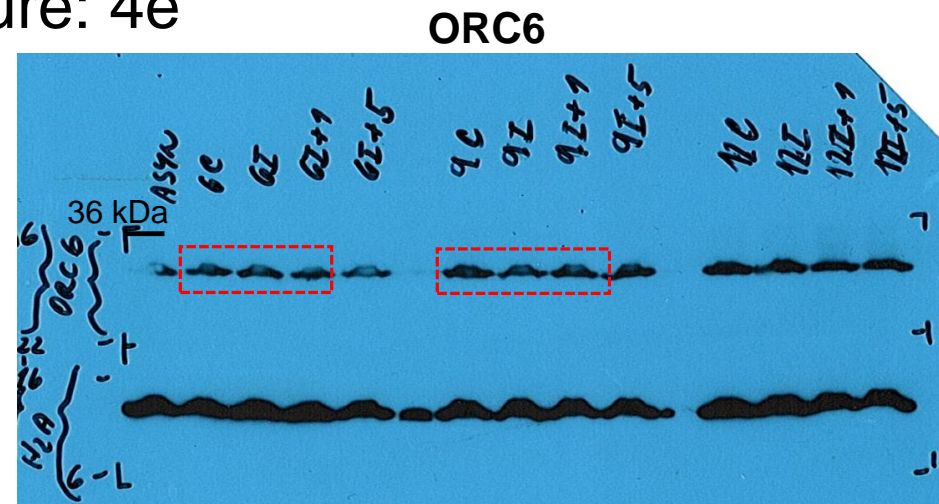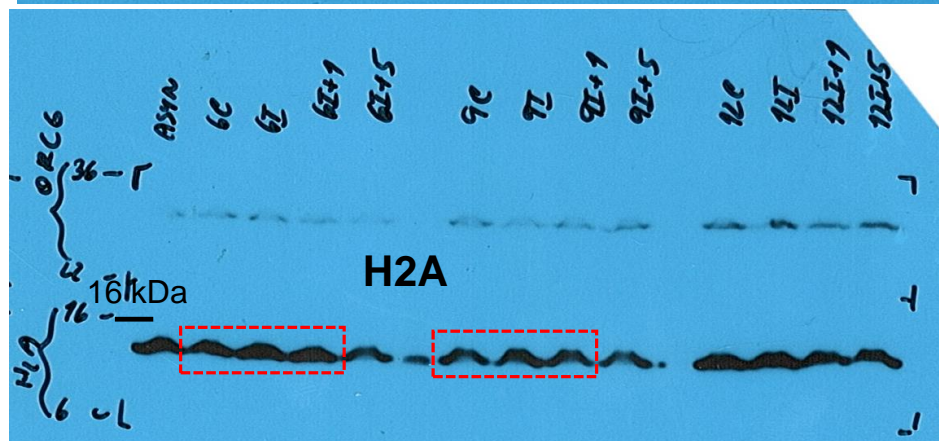

**H2A**  
(Short exposure)

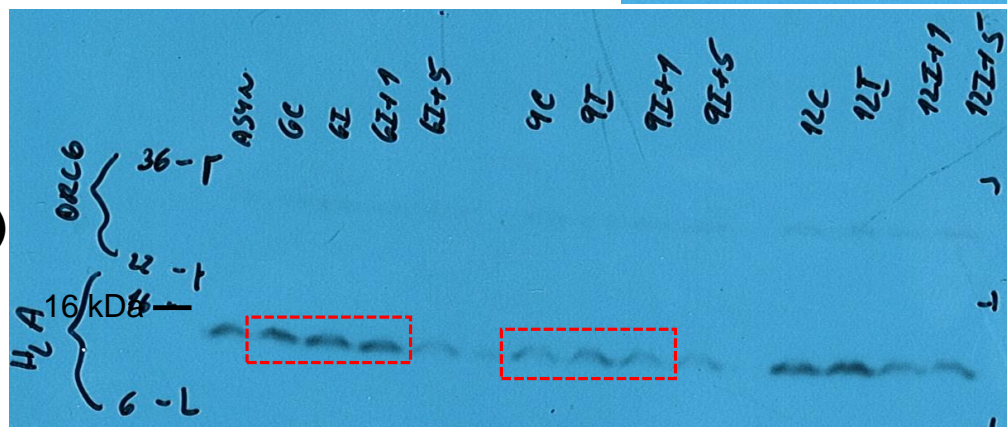

Supplement: Supplementary file 11 — Source Data for Figure 4 [file EMBR-20-e47592-s009.pdf]
